# Spatial Distribution of the Metabolically Active Microbiota within Italian PDO Ewes' Milk Cheeses

**DOI:** 10.1371/journal.pone.0153213

**Published:** 2016-04-13

**Authors:** Ilaria De Pasquale, Raffaella Di Cagno, Solange Buchin, Maria De Angelis, Marco Gobbetti

**Affiliations:** 1 Department of Soil, Plant and Food Science, University of Bari Aldo Moro, Bari, Italy; 2 INRA, UR 342, Technologie et Analyses Laitières, Poligny, France; Medical University of South Carolina, UNITED STATES

## Abstract

Italian PDO (Protected Designation of Origin) Fiore Sardo (FS), Pecorino Siciliano (PS) and Pecorino Toscano (PT) ewes’ milk cheeses were chosen as hard cheese model systems to investigate the spatial distribution of the metabolically active microbiota and the related effects on proteolysis and synthesis of volatile components (VOC). Cheese slices were divided in nine sub-blocks, each one separately subjected to analysis and compared to whole cheese slice (control). Gradients for moisture, and concentrations of salt, fat and protein distinguished sub-blocks, while the cell density of the main microbial groups did not differ. Secondary proteolysis differed between sub-blocks of each cheese, especially when the number and area of hydrophilic and hydrophobic peptide peaks were assessed. The concentration of free amino acids (FAA) agreed with these data. As determined through Purge and Trap (PT) coupled with Gas Chromatography-Mass Spectrometry (PT-GC/MS), and regardless of the cheese variety, the profile with the lowest level of VOC was restricted to the region identified by the letter E defined as core. As shown through pyrosequencing of the 16S rRNA targeting RNA, the spatial distribution of the metabolically active microbiota agreed with the VOC distribution. Differences were highlighted between core and the rest of the cheese. Top and bottom under rind sub-blocks of all three cheeses harbored the widest biodiversity.

The cheese sub-block analysis revealed the presence of a microbiota statistically correlated with secondary proteolysis events and/or synthesis of VOC.

## Introduction

Environmental and technological (e.g., shaping, salting, temperature and time of ripening) drivers impose the taxonomic structure of the cheese microbiota. The microbiota determines the main biochemical changes during ripening, which lead to the unique cheese flavor [1, 2]. Depending on the variety, the distribution of the microbiota may vary between core and surface layers. For the same variety, the microbiota also varies depending on dairies, cheese batches and ripening duration. Intrinsic (availability of substrates and co-factors, presence of inhibitor/activator compounds, pH, and redox potential) and extrinsic factors (oxygen availability, temperature, and relative humidity) [2–6] drive the spatial distribution of microbes within the cheese. Consequently, this distribution determines the growth and function of the microbial community [7, 8], which undoubtedly affects ripening, flavoring, protection, and spoilage of each cheese layer. A few studies have investigated the bacterial distribution and movement in cheeses [8–12].

After milk stirring and coagulation, bacteria are immobilized in the cheese curd according to a relatively uniform but stochastic distribution. Such immobilization affects the spatial repartition of colonies, and creates microscopic environmental niches, that are subjected to fluctuations throughout space and time (ripening) [10]. After manufacture and during cheese ripening, primary starters usually undergo cell disintegration, which, in most of the cases, culminates in the complete lysis [8]. On the contrary, non-starter lactic acid bacteria (NSLAB) and sub-dominant bacteria remain metabolically active, mainly intact and, probably, subjected to a dynamic distribution. In general, lactic acid bacteria and mainly mesophilic NSLAB dominate the core of most the cheese varieties because of their adaptation to the environmental conditions and the efficient enzyme systems [13–15]. Most of the metabolic functions of NSLAB (e.g., fermentation of lactate, citrate, amino-sugars, and glycerol, and catabolism of peptides and amino acids) are decisive for cheese flavor. Primary starters also overlap some of the above metabolisms but, in particular, synthesize aromatic compounds, provide substrates for other microorganisms, and regulate the early growth of the cheese microbiota [7]. Metabolic interactions among microbes also occur at the cheese surface [7, 16]. The cheese surfaces of several varieties show a large eukaryote and prokaryote diversity. Yeast-yeast and yeast-bacterium interactions may affect the establishment of the cheese surface ecosystem [17, 18].

To date, two approaches, which differed for sampling procedure and technique for bacterial identification, are used to describe the spatial distribution of bacteria in cheeses [12]. The first approach is non-destructive. It uses model systems (namely gel cassette) [10] or cryo-sectioning, followed by FISH fluorescence in situ hybridization based on fluorescently labelled oligonucleotide probes [9, 19]. The second and most used approach is destructive. Cheese sections are selected, followed by culture-dependent or -independent methods of identification [8, 11, 20–22]. This approach has evolved to next-generation sequencing technology, being used for describing the temporal and spatial distribution (rind and core) of the microbial population of brine salted Continental-type cheese. For instance, the day timing (morning or afternoon) for cheese manufacture affected the spatial distribution of the microbiota throughout ripening [12]. Drivers for assembling the microbial communities of 137 cheese rinds were excellently elucidated through high-throughput sequencing [23]. None of the above studies has established the relationship between the spatial microbial distribution and proteolysis and synthesis of volatile components of hard-cheese varieties.

Italy is one of the worldwide countries having the largest and most diverse production of cheeses made with cows’, goats’, buffalos’ and, especially, ewes’ milk [24]. Pecorino is the trivial name given to Italian cheeses made with raw or heated ewes’ milk, which are mainly manufactured in the Centre and South Italy according to ancient and unique techniques. Fiore Sardo, Pecorino Siciliano and Pecorino Toscano cheeses are some of the most famous, having the recognition of Protected Designation of Origin (PDO). These cheeses are manufactured using raw milk (except for Pecorino Toscano) and rennet paste, without starter cultures (except for Pecorino Toscano), and, although consumed after various ripening age, they mostly belong to the hard-cheese category. Owing to the large size and the prolonged brine and/or dry salting, these cheese varieties are characterized by a decreasing NaCl gradient from the surface to the center and by an opposite moisture trend, which reflects the values of water activity (*a*_*w*_). These gradients persist for considerable time, thus affecting the spatial region during cheese ripening [24]. Therefore, the structure and growth of the microbiota might be markedly influenced across cheese sections.

This study used the three above Italian PDO ewes’ milk cheeses as model systems to investigate: (i) the spatial gross chemical composition; (ii) the spatial distribution of metabolically active bacteria through pyrosequencing of the 16S rRNA targeting RNA; and (iii) how this distribution reflects on the content of volatile components and cheese proteolysis.

## Material and Methods

### Cheese samples

**Fig 1:**
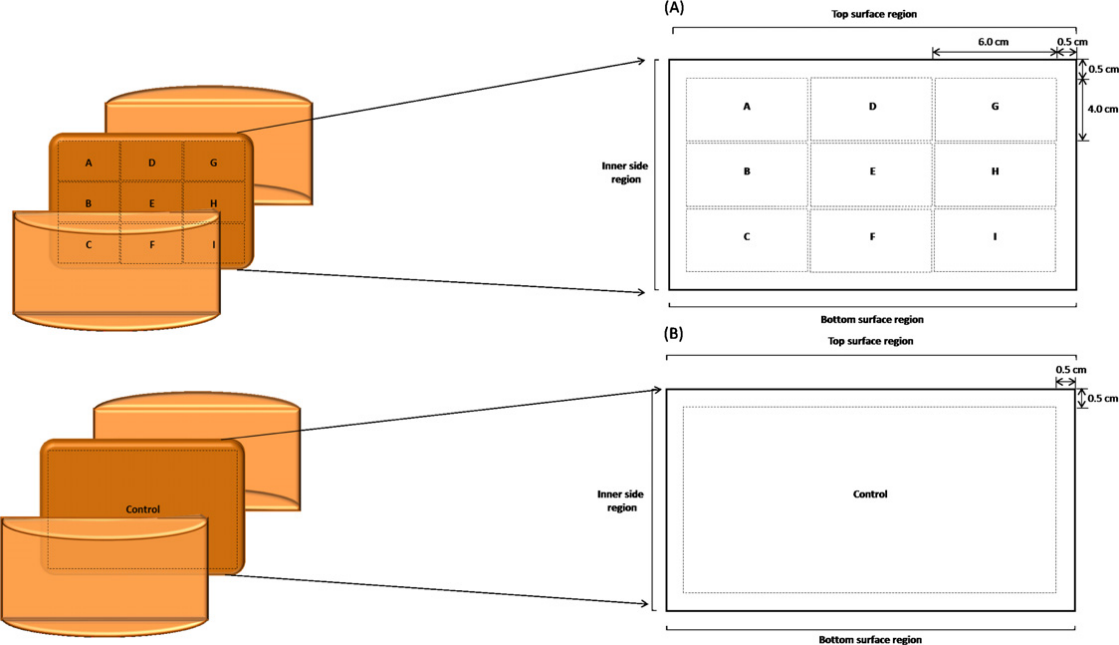
Schematic representation of the cheese sampling procedure. A slice of each cheese was cut into nine sub-blocks identified by the letters A—I. Sub-blocks A, D, and G, and sub-blocks C, F and I were collected from top and bottom surface region, respectively, whereas sub-blocks B and H from inner side region, and sub-block E from the core (A). The whole cheese slice was used as the control (B). Further details were reported in the Material and Methods.

Three batches of Fiore Sardo (FS), Pecorino Siciliano (PS) and Pecorino Toscano (PT) ewes’ milk cheeses were supplied by Sepi Formaggi srl, (Marrublu, Sardinia, Italy), Casa del Formaggio Sant’Anna (Ragusa, Sicily, Italy) and Spadi Enzo (Roccastrada, Tuscant, Italy), respectively. All three dairies have legal permissions to produce the PDO varieties. Fiore Sardo and Pecorino Siciliano were manufactured from raw ewes’ milk and lamb rennet paste was used, whereas Pecorino Toscano was manufactured with pasteurized (73°C for 5 s) ewes’milk and calf rennet paste. Fiore Sardo and Pecorino Siciliano cheese making was without starters. Pecorino Toscano cheese was started with selected autochthonous cultures of *Lactococcus lactis*. The protocol for making Pecorino Siciliano cheese included the initial moulding of the curd in hot whey at 80°C for ca. 20 min. The three cheeses were ripened for ca. 4 months. The average weight of the cheeses was ca. 4.5 kg, with a diameter of 16–22 cm and height 13–16 cm. In order to investigate the microbial spatial distribution and its effects on biochemical events (proteolysis and volatile compounds profile), each cheese was cut along the vertical axis to obtain two symmetrical halves, and three vertical slices were taken from one half. Each slice was cut into nine sub-blocks identified by the letters A—I (Fig 1A). Sub-blocks A, D, and G, and sub-blocks C, F and I were collected from top and bottom surface region, respectively (ca. 0.5 cm from rind), whereas sub-blocks B and H from the inner side region (ca. 0.5 cm from rind) and sub-block E from core. Top and bottom regions of the cheese were arbitrarily indicated only to facilitate the description of cheese sub-blocks. This sampling procedure reflected the consideration that the general characterization of cheese microbiota and related biochemical events refer to the entire cheese ecosystem and the whole cheese slice (control) was considered for comparison with cheese sub-blocks (Fig 1B).

All the results from compositional, microbiological and biochemical analyses were the average of 3 batches for each variant of cheese; each batch was analyzed in triplicate (total 9 samples analyzed for each cheese variant). According to previous studies [25–28] data from pyrosequencing result from the pooling of three cDNA samples, corresponding to the three batches for each sub-block or for the control. All samples were transported to the laboratory into thermal plastic bag under refrigerated condition (ca. 4°C), and analyzed immediately (microbiological analysis) or frozen (–80°C) (biochemical analysis and extraction of total RNA).

### Compositional, microbiological, and biochemical analyses

Samples of cheese were analyzed for protein [29], fat [30], moisture (oven drying at 102°C) [31] and salt [32]. The pH was measured by Foodtrode electrode (Hamilton, Bonaduz, Switzerland).

Microbiological analyses were carried out as described previously [33]. Twenty grams of sample were homogenized with 180 ml of sterile sodium citrate (2%, [wt/vol]) solution. Presumptive mesophilic lactobacilli and lactococci were enumerated onto MRS and M17 agar (Oxoid, Basingstoke, Hampshire, UK), respectively, under anaerobiosis at 30°C for 48 h. Presumptive thermophilic streptococci were enumerated onto M17 agar (Oxoid), under anaerobiosis at 42°C for 48 h. Enterococci were counted onto Slanetz & Barteley Agar (Oxoid) at 37°C for 48 h. Except for enterococci, the media for plating bacteria were supplemented with cycloheximide at 0.1 g/l.

The pH 4.6-insoluble and -soluble nitrogen fractions of the cheeses were analyzed by urea polyacrylamide gel electrophoresis (Urea-PAGE) and reverse phase high pressure liquid chromatography (RP-HPLC), as described by Andrews et al. [34], and Gobbetti et al. [35], respectively. Total and individual free amino acids (FAA) from the pH 4.6-soluble fraction were determined by a Biochrom 30 series Amino Acid Analyzer (Biochrom Ltd., Cambridge Science Park, UK), as described by Di Cagno et al. [36].

### Determinations of neutral volatile components and volatile free fatty acids

Neutral volatile components (VOC) were determined by Purge and Trap (PT) coupled with Gas Chromatography-Mass Spectrometry (PT-GC/MS). Prior to PT, 3 g of cheese were mixed with 27 g of UHQ deodorized water, with an ultra-turrax during 4 x 40 s separated by 10 s rest, in a glass flask with a narrow neck plunged into ice. Ten ml of this suspension were placed in a glass extractor connected to the PT apparatus (Tekmar 3000, Agilent Instruments, NY, USA). Extraction was performed by helium at a flow rate of 40 ml/min on a tenax trap at 37°C. Trap was desorbed at 225°C and injection used a cryo-concentrator at -150°C. The chromatograph (6890 Agilent Instruments) was equipped with a DB5-like capillary column (RTX5 Restek, Agilent Instruments), 60 m length, 0.32 μm internal diameter, and 1 μm thickness. The helium flow rate was 2 ml/min, the oven temperature was 40°C during the first 6 min and then was increased at 3°C/min to 230°C. The mass detector (MSD5973, Agilent Instruments) was used in electronic impact at 70 eV and in scan mode, from 29 to 206 atomic mass. Quantification of compounds was expressed in arbitrary units of area.

### Extraction of total RNA

Because the diversity of metabolically active microbiota has relevant repercussions on food ecosystems(e.g., fate of starter or adjunct cultures versus microbial contaminants), high throughput sequencing from RNA data was adopted as it may provide a more complete description of the microbiota [37]. Besides, this approach may be useful especially when, cheese manufactured with pasteurized milk and started with starter cultures were used (e.g., Pecorino Toscano). Total RNA was extracted using the RiboPureTM—Bacteria Kit (Ambion RNA, Life Technologies Co., Carlsbad, CA, USA), according to the manufacturer’s instructions. Quality control of RNA was checked through agarose gel electrophoresis. The RNA concentration was measured in a NanoDrop ND-1000 spectrophotometer (NanoDrop Technologies, Rockland, DE). In order to remove DNA, the purified RNA (100 ng) (final volume, 20 μl) was incubated at 42°C for 2 min in 2 μl of gDNA Wipeout Buffer 7X (QuantiTect Reverse Transcription Kit, Qiagen srl, Milan, Italy) and RNase-free water (final volume, 14 μl). The cDNA was obtained by the QuantiTect Reverse Transcription Kit (Qiagen), according to the manufacturer’s instructions. All reactions were set up in a Rotor Gene 6000 instrument (Corbett Life Science, New South Wales, Australia) equipped with a 36-well reaction rotor.

### Pyrosequencing and data analyses

Three cDNA samples, corresponding to the three batches for each sub-block or for the whole cheese slice, were pooled and used for 16S based bacterial diversity analysis. Bacterial diversity was assessed via pyrosequencing on a 454 FLX Sequencer (454 Life Sciences, Branford, CT, USA) and was performed by Research and Testing Laboratories (Lubbock, TX), according to standard laboratory procedures. Primers targeting the V1–V3 region (*Escherichia coli* position 27–519, forward 28F: GAGTTTGATCNTGGCTCAG and reverse 519R: GTNTTACNGCGGCKGCTG) of the 16S rRNA gene [38] were used. Pyrosequencing procedures were carried out based upon RTL protocols http://www.researchandtesting.com (Research and Testing Laboratories, Lubbock, TX).

### Bioinformatics

Sequence data for each cheese variant were processed using Research and Testing Laboratory’s in-house pipeline, described at http://www.researchandtesting.com/docs/Archive/Data_Analysis_Methodology-2.2.3.pd. Briefly, sequences were grouped using their barcodes and any sequence that contained a low quality barcode or that failed to be at least half the expected amplicon length (or 250 bp, whichever was shortest) was removed from the data pool. Sequences that passed the quality filter were denoised using an algorithm based on USEARCH pipeline [39], and then checked for chimeras using UCHIME [40]. Finally, sequence data were separated into operational taxonomic units (OTUs) at 97% similarity using a USEARCH and all OTUs were used for classification by using UBLAST global alignment against a custom 16S database comprised of well characterized sequences from nr/nt. The output was then analyzed using a internally developed Python pipeline that parses the assigned taxonomic information to create the final analysis files. Alpha- and beta-diversities were evaluated by QIIME, as recently described [41].

### Statistical analyses

Data were subjected to one-way ANOVA, and pairwise of treatment means was achieved by Tukey’s procedure at P<0.05, using the statistical software Statistica for Windows (Statistica 7.0, per Windows). Data of VOC and correlations between the abundance of operational taxonomic units (OTUs) and total free amino acid (FFA), concentration of Ser, Glu, Asp, and Arg, number of hydrophilic peaks of the pH 4.6-soluble nitrogen fractions, and volatile components (arbitrary units of area) were subjected to permutation analysis using PermutMatrix [42]. Only the positive correlations with a P<0.05, FDR<0.05 and r>0.7 were considered.

### Nucleotide sequence accession number

All the sequencing data were deposited at the Sequence Read Archive of the National Center for Biotechnology Information (PRJNA286758).

## Results

### Compositional and microbiological analyses

S1 Table shows the main chemical composition of Fiore Sardo (FS), Pecorino Siciliano (PS) and Pecorino Toscano (PT) PDO ewes’ milk cheeses. As expected, the highest value of moisture was found in the core (sub-block E) (FS, 25.2 ± 1.3%; PS, 25.0 ± 1.1%; and PT, 20 ± 1.2%) and adjacent D (FS, 18.2 ± 1.2%; PS, 22.0 ± 1.2%; and PT, 18 ± 1.3%) and F (FS, 19.2 ± 1.1%; PS, 23.0 ± 0.9%; and PT, 17 ± 1.0%) cheese sub-blocks. The moisture of all the other sub-blocks was uniform and significantly (P<0.05) lower. The concentration of salt, fat and protein inversely followed the moisture trend. The values of pH did not significantly (P>0.05) differ among sub-blocks. The few exceptions were the core E of PS cheese (5.9 ± 0.3), which was slightly but significantly (P<0.05) higher than the other sub-blocks, and the top surface region under rind (sub-blocks A, D and G) of PT cheese that showed a higher value of pH (ca. 6.0) than the other sub-blocks (ca. 5.6). The gross composition of the control samples was almost the average of the individual values that were found into the sub-blocks (S1 Table).

The cell numbers of the principal microbial groups from the three batches of each cheese variety did not significantly differ (P>0.05). Control samples of FS, PS and PT cheeses contained presumptive mesophilic lactobacilli and lactococci, thermophilic streptococci and enterococci in the ranges of 6.2–8.0, 6.0–8.4, 6.2–7.6 and 4.7–6.3 log CFU/g, respectively (Table 1). Mesophilic lactobacilli were the highest (8.0 ± 0.2 log CFU/g) in PS cheese. PT cheese contained the highest number of mesophilic lactococci (8.4 ± 0.2 log CFU/g). No significant (P>0.05) differences were found between sub-blocks of each cheese regarding cell counts of the above microbial groups. The only exception was core E and adjacent sub-blocks D and F of PS cheese, which had a slightly lower (P<0.05) content of mesophilic lactobacilli than the other sub-blocks.

**Table 1:** Cell numbers (log CFU/g)^*^ of various microbial groups in Fiore Sardo, Pecorino Siciliano and Pecorino Toscano cheeses.

Microbial group	Control^§^	A	D	G	B	E	H	C	F	I
	**Fiore Sardo**									
Mesophilic lactobacilli	7.1±0.2^c^	7.6±0.1^a^	7.1±0.4^c^	7.1±0.1^c^	7.2±0.3^c^	7.7±0.2^a^	7.6±0.1^a^	7.4±0.1^b^	7.0±0.1^c^	7.3±0.3^b^
Mesophilic lactococci	8.2±0.2^c^	8.5±0.3^b^	8.7±0.1^b^	8.3±0.1^c^	8.4±0.1^b^	8.7±0.3^a^	8.2±0.1^c^	8.7±0.2^a^	8.8±0.1^a^	8.4±0.4^b^
Thermophilic streptococci	7.3±0.1^a-b^	6.9±0.2^c^	7.0±0.2^b^	7.2±0.2^a-b^	7.5±0.3^a^	7.1±0.1^b^	6.8±0.4^c^	7.0±0.1^b^	6.9±0.3^c^	7.2±0.2^a-b^
Enterococci	6.3±0.1^a-b^	5.8±0.2^c^	6.2±0.1^a-b^	5.9±0.1^c^	5.5±0.2^d^	6.3±0.4^a^	5.4±0.3^d^	5.5±0.3^d^	6.3±0.5^a^	5.5±0.1^d^
	**Pecorino Siciliano**									
Mesophilic lactobacilli	8.0±0.2^a^	8.2±0.1^a^	7.8±0.2^b^	8.1±0.2^a^	7.9±0.3^b^	7.3±0.1^d^	7.5±0.4^c^	8.0±0.4^a-b^	8.0±0.2^a^	7.8±0.4^b^
Mesophilic lactococci	6.0±0.2^b^	5.8±0.4^b-c^	5.6±0.2^c^	6.1±0.1^b^	5.8±0.1^c^	5.5±0.1^c^	6.4±0.3^a^	6.3±0.1^a^	6.0±0.2^b^	5.9±0.2^b^
Thermophilic streptococci	7.6±0.1^c^	7.1±0.1^d^	7.6±0.1^c^	7.7±0.2^b^	7.2±0.1^d^	7.9±0.3^a^	7.7±0.4^a^	7.3±0.2^c-d^	7.4±0.0^c-d^	7.2±0.1^d^
Enterococci	6.0±0.1^b^	6.0±0.2^b^	5.9±0.3^b^	6.1±0.2^a-b^	6.3±0.4^a^	5.5±0.1^c^	5.9±0.2^b^	6.5±0.1^a^	6.2±0.4^a^	6.3±0.4^a^
	**Pecorino Toscano**									
Mesophilic lactobacilli	6.2±0.3^a^	6.3±0.1^a^	6.5±0.2^a^	6.1±0.1^b^	5.9±0.2^b^	6.4±0.1^a^	6.3±0.2^a^	5.7±0.1^c^	5.8±0.3^b^	5.8±0.2^b^
Mesophilic lactococci	8.4±0.2^b^	8.1±0.1^c^	8.0±0.2^c^	8.1±0.32^e^	8.5±0.3^b^	8.7±0.3^a^	8.3±0.1^d^	8.6±0.1^b^	8.6±0.1^b^	8.4±0.3^b^
Thermophilic streptococci	6.2±0.1^a^	5.9±0.2^b^	5.6±0.1^c^	6.1±0.3^a^	5.4±0.1^c^	5.8±0.2^b^	6.1±0.4^a^	5.7±0.3^b^	6.2±0.1^a^	5.9±0.1^b^
Enterococci	4.7±0.4^a-b^	4.8±0.2^a-b^	4.6±0.1^c^	4.6±0.1^c^	4.2±0.1^d^	4.5±0.2^c^	4.9±0.4^a^	4.5±0.3^b^	4.5±0.4^b^	4.3±0.1^d^

Data in the same row with different superscript letters (a-f) are significantly different (P<0.05).

*Mean values ± standard deviations for three batches of each variant of cheese, analyzed in triplicate.

^§^Slice of each cheese was cut into nine sub-blocks identified by the letters A—I. Sub-blocks A, D, and G, and sub-blocks C, F and I were collected from top and bottom surface region, respectively, whereas sub-blocks B and H from inner side region, and sub-block E from the core. The whole cheese slice was used for the comparison with cheese sub-blocks Control. Further details were reported in the Material and Methods and in Fig 1.

### Proteolysis

**Fig 2:**
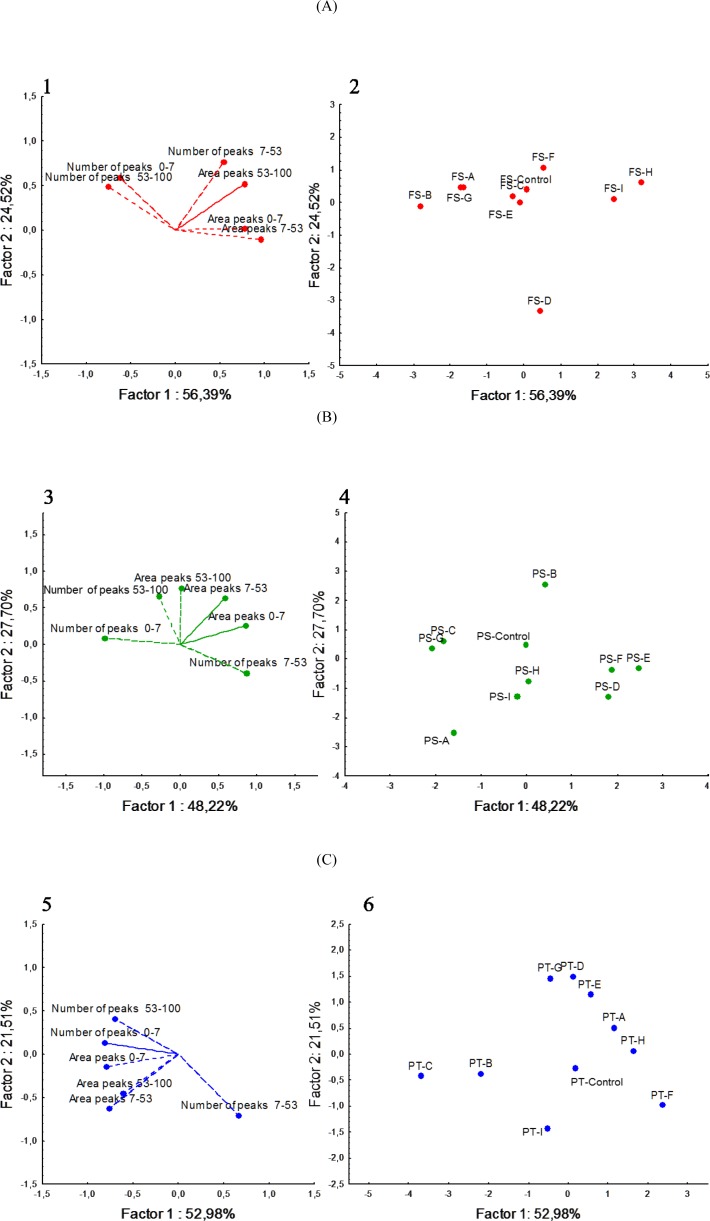
Score plot and loading plot of the first and secondary principal components based on area and number peaks obtained from reverse phase high pressure liquid chromatography (RP-HPLC). Score plot and loading plot (panels 1, 3, 5) of the first and secondary principal components (PC) (panels 2, 4, 6) after PC analysis based on area and number peaks obtained from reversed-phase high-protein liquid chromatograms of the pH 4.6-soluble fractions of Fiore Sardo (A), Pecorino Siciliano (B) and Pecorino Toscano (C). Nine sub-blocks are identified by the letters A—I. Sub-blocks A, D, and G, and sub-blocks C, F and I were collected from top and bottom surface region, respectively, whereas sub-blocks B and H from inner side region, and sub-block E from the core. The whole slice was the control Control. Further details were reported in the Material and Methods and in Fig 1.

The urea-PAGE electrophoretograph of pH 4.6-insoluble and -soluble nitrogen fractions did not show differences between sub-blocks of each cheese (data not shown). Overall, α_s_1-casein (CN) was completely degraded in FS and PT, but not in PS cheese. β-CN persisted to the end of ripening for all three cheeses, and formation of protein bands with low electrophoretic mobility, which presumably corresponded to γ-CN, was evident and indicated plasmin activity. The urea-PAGE electrophoretograph of the pH 4.6-soluble fractions showed characteristic polypeptide bands for all three cheeses. Complementary information emerged by RP-HPLC analysis (S1 Fig). The number and the area of peaks, which were recognized and matched visually with the Unicorn program (Amersham Biosciences), varied (quantitatively and qualitatively) between sub-blocks of FS, PS and PT cheeses (13–19, 13–15 and 14–20 peaks, respectively) (S1 Fig). Principal component analysis (PCA) was applied to RP-HPLC data (number and the area of hydrophobic and hydrophilic peptide peaks) (Fig 2). The 2 PC for FS, PS and PT cheeses explained, respectively, 80.9, 75.9 and 74.5% of the total variance. The sub-block D of FS cheese showed the lowest concentration of peptides, whereas the other sub-blocks occupied an almost uniform and separated zone of the plane. For PS cheese, the highest concentration of peptides was found in sub-blocks D, E and F, which were grouped together in the plane. Sub-blocks B, C, I and F of PT cheese showed the highest concentration of peptides, whereas the other sub-blocks occupied separate zones of the plane. The whole cheese slice (control) of all three semi-hard cheeses was located in the center of the plane.

**Fig 3:**
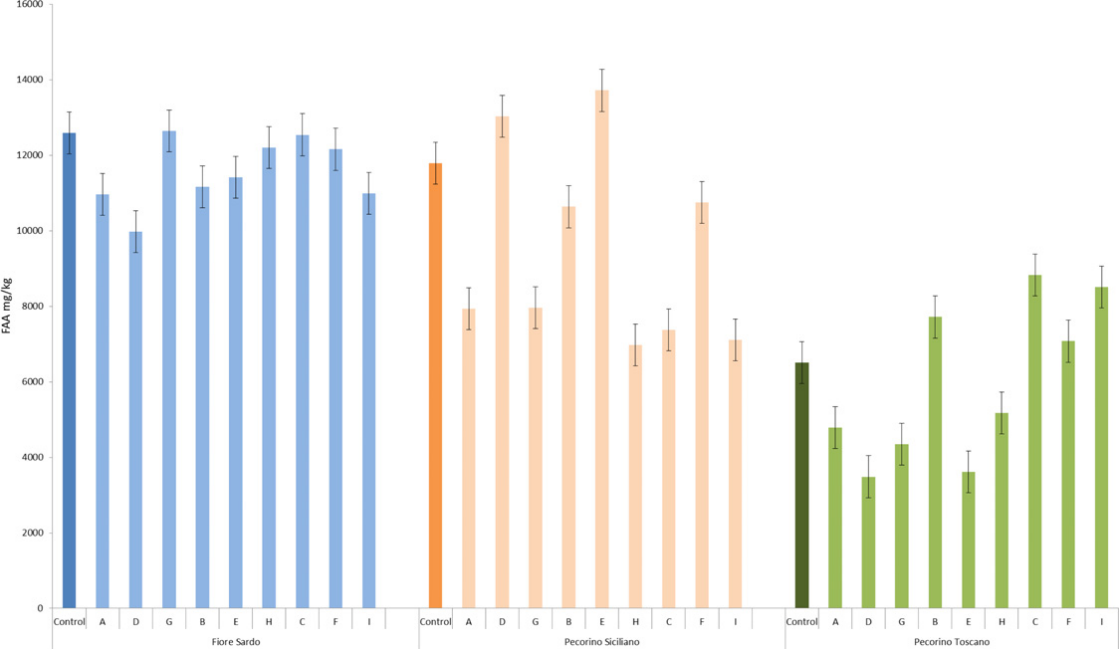
Concentrations of total free amino acids (FAA) (mg/kg) identified in Fiore Sardo, Pecorino Siciliano and Pecorino Toscano cheeses. Nine sub-blocks are identified by the letters A—I. Sub-blocks A, D, and G, and sub-blocks C, F and I were collected from top and bottom surface region, respectively, whereas sub-blocks B and H from inner side region, and sub-block E from the core. The whole slice was the control Control. Further details were reported in the Material and Methods and in Fig 1.

These results agreed with the concentration of total free amino acids (FAA) (Fig 3 and S2 Table). Regarding control samples, the concentration of FAA followed the order FS—PS (12590.8 ± 72.5 and 11788.2 ± 64.9 mg/kg), and PT (6507.3 ± 32.1 mg/kg) cheeses. Within FS cheese, the sub-block D had the lowest concentration of FAA (9977.9 ± 32.4 mg/kg). Sub-blocks D (13033.2 ± 11.4 mg/kg), E (13715 ± 75.6 mg/kg) and F (10750.7 ± 62.9 mg/kg) of PS cheese showed the highest level of FAA. Sub-blocks I (8510.2 ± 72.8 mg/kg), C (8830.1 ± 63.7 mg/kg), B (7714.5 ± 33.1 mg/kg) and F (7075 ± 63.3 mg/kg) of PT cheese contained the highest levels of FAA. Overall, the FAA found at the highest concentrations (>100 mg/kg) were Ser, Glu, Leu, Arg, and Phe. FS and PS cheeses showed Val and Asp at the highest concentrations, respectively.

### Volatile components

**Fig 4:**
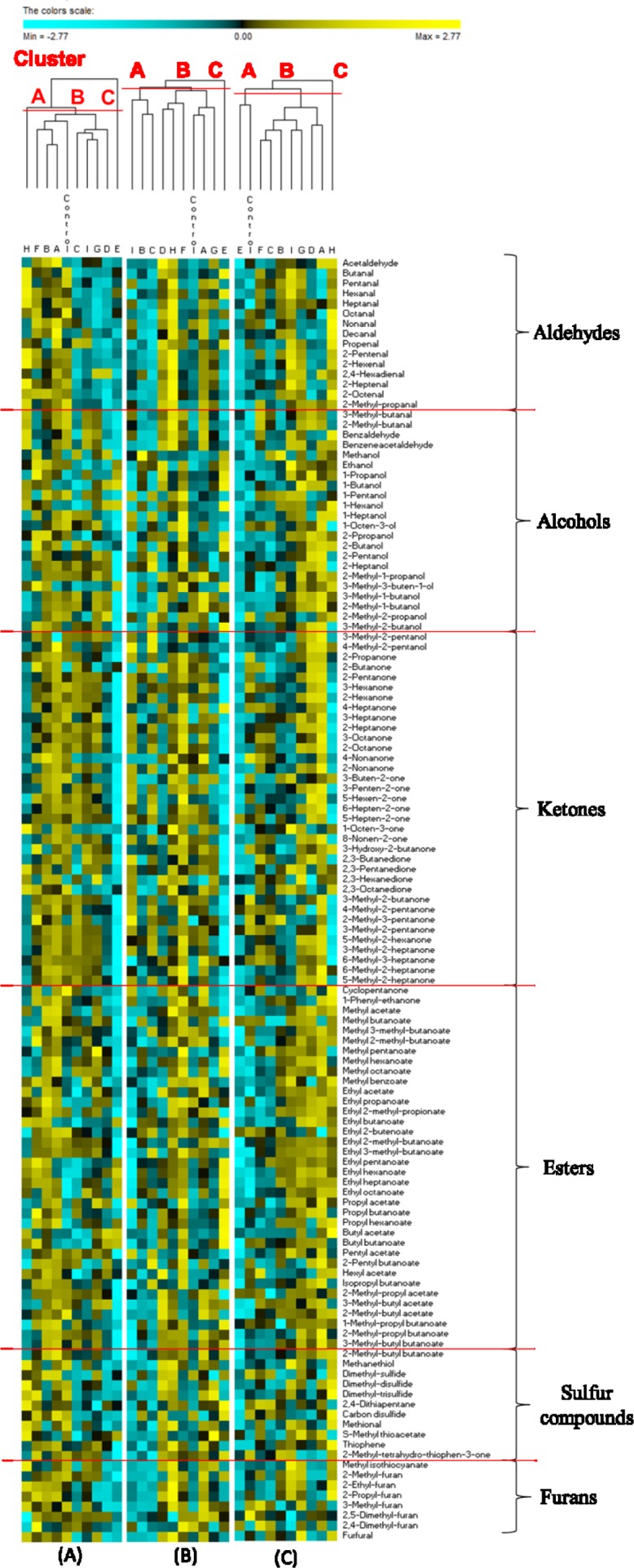
Permutation analysis of volatile components. Concentrations of volatile components (arbitrary units of area) identified from Fiore Sardo (A), Pecorino Siciliano (B), and Pecorino Toscano (C) cheese. Euclidean distance and McQuitty’s criterion (weighted pair group method with averages) were used for clustering. The colors correspond to normalized mean data levels from low (blue) to high (yellow). The color scale, in terms of units of standard deviation, is shown at the top. Nine sub-blocks are identified by the letters A—I. Sub-blocks A, D, and G, and sub-blocks C, F and I were collected from top and bottom surface region, respectively, whereas sub-blocks B and H from inner side region, and sub-block E from the core. The whole slice was the control. Further details were reported in the Material and Methods and in Fig 1.

A total of 152 neutral volatile components (VOC) were commonly identified in the three semi-hard cheeses by PT-GC/MS. No significant (P>0.05) differences were found between the three batches of each cheese analyzed. The levels of 125 VOC significantly (P<0.05) differentiated sub-blocks of each cheese (S3 Table and Fig 4). The sub-block E of FS cheese contained the lowest level of VOC, being represented by some alcohols (primary alcohols), esters (ethyl and butyl esters) and S-methyl thioacetate at the highest levels. Top (D and G) and bottom (C and I) sub-blocks contained high levels of several secondary and branched alcohols and, especially, ketones (all sorts), and low levels of aldehydes and sulfur compounds. On the contrary, these latter compounds were found at particularly high level in H sub-block. Globally, B, A and F sub-blocks were characterized by high or medium levels of all the classes of compounds. Almost all VOC, which were found in sub-blocks, were also detectable in the control sample of FS cheese. The core E of PS cheese also showed the lowest level of VOC (mainly aldehydes and ketones). C, I, and B sub-blocks had low levels of aldehydes, sulfur compounds and furans. C and I had high levels of ketones. The VOC profile of control cheese was almost similar to that of sub-blocks C, I and B. The other sub-blocks showed the largest amount of almost all VOC: aldehydes, branched esters, sulfur compounds, furans, ketones for A, F, and H, and branched alcohols for A and D sub-blocks. For PT cheese, the core E showed the lowest levels of all the VOC. The top under rind sub-blocks (A, D and G) and I sub-block were the richest in VOC, mainly alcohols (e.g., 1-octen-3-ol), ketones (e.g., 2-heptanone) and esters (e.g., ethyl acetate), followed by the inner side (H) sub-block, where almost all the compounds (except for ketones) were at high levels. Globally, sub-blocks C, F and B, and the control showed medium levels of VOC, being ketones (e.g., 2-methyl-3-pentanone) and aldehydes (e.g., butanal) the highest. The permutation analysis based on VOC (Fig 4) distributed the sub-blocks of each cheese into three major clusters (A, B and C). The core E of all three cheeses was singly grouped. The top and bottom under rind sub-blocks almost grouped in the same clusters, being the H sub-block that most diverse in FS and PT cheeses.

### 16S rRNA gene pyrosequencing

A total of 50,290, 34,932 and 57,640 raw sequence reads of 16S rRNA gene amplicons were obtained for FS (average length 475 bp), PS (average length 465 bp) and PT (average length 472 bp) cheeses, respectively. For all the three cheeses, the highest values of the Shannon diversity index were always found in top and bottom under rind sub-blocks (S4 Table). An almost similar trend was found for Chao1 richness. The Good’s estimated sample coverage (ESC) was above 99% for all the samples, indicating a satisfactory description of the microbial diversity. Metabolically active bacteria were also analyzed using three phylogeny-based beta-diversity measures (S2 and S3 Figs). The principal coordinate analysis (PCoA) based on the unweighted UniFrac distance matrix differentiated the cores E of PS and PT cheeses, which were characterized by low variability. Except for C, I and B, all sub-blocks and control of FS cheese grouped together (S2 Fig).

RNA from sub-blocks of FS and PS cheeses included mainly *Firmicutes* (99–100 and 97.8–100%, respectively) and *Bacteroidetes* at very low incidence (0.07–0.10%, respectively). *Firmicutes* was also the main phylum detected into sub-blocks of PT cheese (91.3–99.9%), followed by *Actinobacteria* (0.10–5.84%). *Proteobacteria* (0.06–2.59%) and *Bacteroidetes* (0.02–0.29%) were found only in the top under rind sub-blocks (A, D and G).

**Fig 5:**
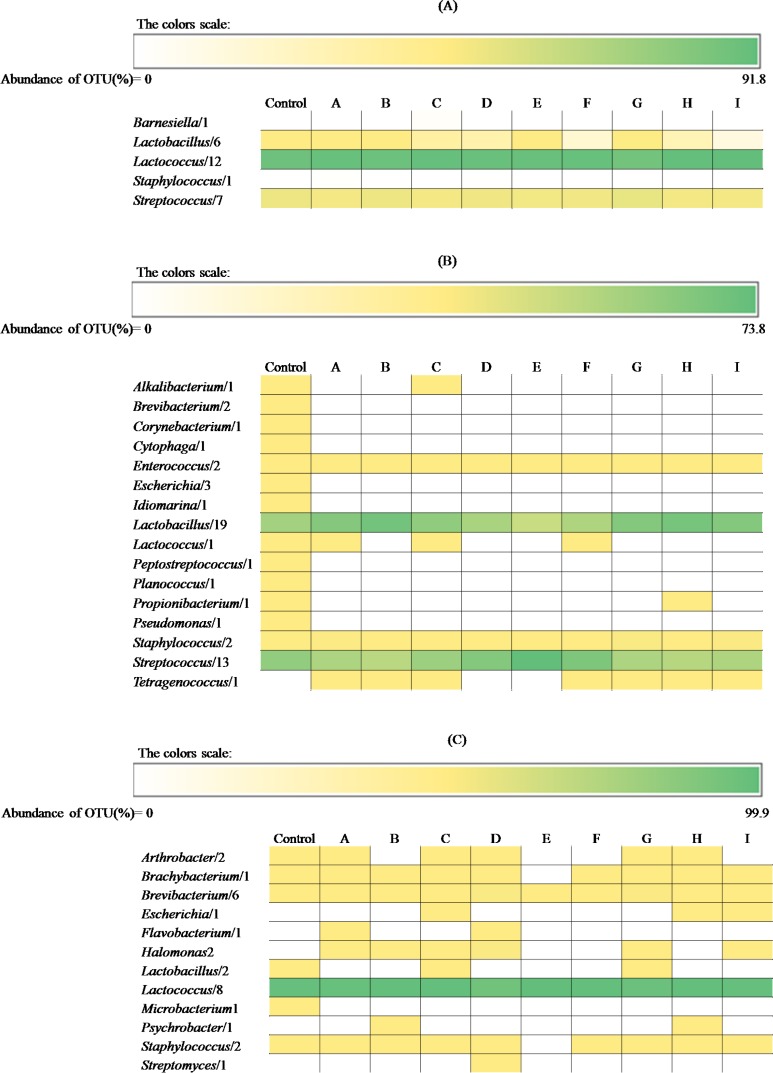
Abundance and number of operational taxonomic units (OTUs) assigned at genus level occurring in Fiore Sardo, Pecorino Siciliano, and Pecorino Toscano cheeses. Distribution of the OTUs assigned at genus level occurring in Fiore Sardo (A), Pecorino Siciliano (B), and Pecorino Toscano (C) cheese. Nine sub-blocks are identified by the letters A—I. Sub-blocks A, D, and G, and sub-blocks C, F and I were collected from top and bottom surface region, respectively, whereas sub-blocks B and H from inner side region, and sub-block E from the core. The whole slice was the control. Further details were reported in the Material and Methods and in Fig 1.

The total number of OTUs was 87, of which 29, 51 and 29 were detected in FS, PS and PT, respectively. At genus level, the number of identified OTUs of sub-blocks and control samples varied depending on the cheese: 5, 16 and 12 OTUs for FS, PS and PT, respectively. *Lactococcus* (ranging from 82.4 to 91.8%), *Streptococcus* (7.8 to 14.9%) and *Lactobacillus* (0.4 to 2.6%) (Fig 5A) were the main genera found in FS cheese. Their abundance did not differ between sub-blocks. The only exception was *Lactobacillus*, which was slightly lower in D, F, I, and H sub-blocks. The same three OTUs at almost the same abundance were identified in the control sample (86.2, 11.9 and 1.9 respectively). Only 7 over the 16 OTUs identified for PS cheese had relative abundances higher than 0.5% in at least one sample (Fig 5B). Their abundance varied within sub-blocks. *Lactobacillus* was the lowest in the core E (26.0%), followed by adjacent top (D) (40.5%) and bottom (F) (38.6%) under rind sub-blocks. The values for all the other sub-blocks ranged from 52.2 to 66.3%. An opposite abundance was found for *Streptoccocus*. The corresponding OTU was the highest in the core E (73.8%), followed by sub-blocks D (59%) and F (60.8%). The values for all the other sub-blocks were 32.7–47.1%. *Enterococcus* and, especially, *Staphylococcus* (*Firmicutes* phylum) were manly found in the top (A and G) and bottom (C and I) under rind, and inner side (B and H) sub-blocks (0.1–0.7% and 0.1–2.4%, respectively). *Tetragenococcus* was found only in the top (A and G) and bottom (C and I) under rind, and inner side (B and H) sub-blocks (0.02 to 0.76%). The control of PS cheese had an incidence almost intermediate with respect to the above genera. Several sub-dominants genera (less than 0.4%), belonging to *Actinobacteria*, *Firmicutes* (*Planococcus*, *Alkalibacterium*, *Enterococcus*, *Lactococcus*, *Peptostreptococcus*) and *Proteobacteria* were identified only in the control cheese. Regardless the sub-block, the bacterial profile of PT cheese showed high abundance of *Lactococcus* (90.9 to 99.9%) (Fig 5C). Only *Lactococcus* dominated the core E. *Brachybacterium* (0.02 to 0.9%), *Arthrobacter* (0.05 to 0.2%) and, especially, *Brevibacterium* (0.1 to 4.4%) were found in the top and bottom under rind and inner side sub-blocks. Relatively high incidence of *Halomonas* was found in the A, D and G sub-blocks (0.7, 2.6 and 0.1%, respectively). *Lactococcus* dominated also the control sample (98.3%).

Number and abundance of OTUs from RNA samples are reported in Table 2, with taxonomic details up to species level when such assignment was possible. Apart from the sub-block, *Lactococcus lactis* (from 82.4 to 91.8%; number of OTUs from 5 to 9) and *Streptococcus thermophilus* (from 7.8 to 14.9%; number of OTUs from 5 to 9) were the most abundant species in FS cheese, followed by *Lactobacillus* sp. (from 0.3 to 2.1%; number of OTUs of 1). This latter was found at the highest incidence in the top under rind sub-block G. *Lactobacillus parabuchneri*, which ranged from 0.024 (number of OTUs 1) (sub-block D) to 0.3% (1) (core E), and *Lactobacillus brevis*, from 0.02 (1) (sub-block C) to 0.32% (1) (sub-block G), flanked the above species. The species abundance of PS cheese depended on the sub-block. Compared to all sub-blocks, the core E and the two adjacent D and F sub-blocks contained *Sc*. *thermophilus* as the most abundant species (72.3, 48.3 and 44.5%, respectively) (from 2 to 3), followed by *Lactobacillus delbrueckii* (10.9, 7.2 and 8.2%, respectively) (from 1 to 3) and *Lactobacillus helveticus* (0.14, 0.16 and 0.17%, respectively) (1). The abundance of *Lactobacillus* sp. was opposite (ranging from 51.3 to 58.8%) (from 1 to 2). The control showed an incidence almost intermediate for the above species. Regardless the sub-block, *Lc*. *lactis* was the most abundant species found in PT cheese (from 90.9 to 99.9%) (from 5 to 7). *Brevibacterium* sp., *Brachybacterium* sp. and *Halomonas variabilis* were found at the highest incidence in the top under rind sub-blocks (A, D and G) (number of OTUs of 4, 1 and 2, respectively). *Lc*. *lactis* was the most abundant species found in PT control cheese.

**Table 2:** Operational taxonomic unit (OTUs) occurring at 0.1% abundance in at least one sample and related number, assigned to the species level when such assignment was possible of Fiore Sardo, Pecorino Siciliano and Pecorino Toscano cheeses.

Taxon	Abundance of OTU(%) / Number of OTU^‡^									
	**Fiore Sardo**^**†**^									
	**Control** ^§^	**A**	**D**	**G**	**B**	**E**	**H**	**C**	**F**	**I**
**Number of OTUs**	17	19	18	15	15	19	16	17	19	13
*Lactobacillus brevis*	0.06/1	0.3/1	0.07/1	0.32/1	0	0.03/1	0.08/1	0.02/1	0.02/1	0
*Lactobacillus parabuchneri*	0.33/1	0	0.024/1	0.1/1	0.1/1	0.3/1	0.05/1	0.07/1	0.02/1	0.09/1
*Lactobacillus* sp.	1.5/1	1.4/1	0.8/1	2.1/1	1.6/1	1.4/1	0.7/1	0.9/1	0.4/1	0.3/1
*Lactococcus lactis*	86.2/7	89.6/9	88.5/7	82.4/5	86.9/7	89.3/6	90.8/7	90.6/7	89.7/8	91.8/7
*Streptococcus thermophilus*	11.9/6	8.6/6	10.5/6	14.9/5	11.4/6	8.9/9	8.4/6	8.2/6	9.7/7	7.8/4
	**Pecorino Siciliano**^**†**^									
**Number of OTUs**	31	18	24	23	21	23	24	23	27	21
*Brevibacterium* sp.	0.4/2	0	0	0	0	0	0	0	0	0
*Cytophaga* sp.	0.1	0	0	0	0	0	0	0	0	0
*Enterococcus faecalis*	0.07	0.1	0.08	0.02	0.03	0	0.03	0.01	0.01	0.1
*Enterococcus faecium*	0.06	0.4	0.6	0.3	0.3	0.026	0.16	0.16	0.26	0.26
*Escherichia coli*	0.2	0	0	0	0	0	0	0	0	0
*Escherichia* sp.	0.2	0	0	0	0	0	0	0	0	0
*Idiomarina* sp.	0.4	0	0	0	0	0	0	0	0	0
*Lactobacillus brevis*	0.2/1	0.04/1	0.5/1	0	0.03/1	0.2/1	0.4/1	0	0.2/1	0.3/1
*Lactobacillus buchneri*	0.2	0	0	0	0	0.09	0.03	0	0	0
*Lactobacillus coryneformis*	0.5/1	0	0.08/1	0	0.03/1	0.6/1	0.9/1	0.02/1	0.6/3	0.1/1
*Lactobacillus crustorum*	0	0	0.3	0.05	0.3	0.8	0.06	0.1	0.2	0.02
*Lactobacillus delbrueckii*	14.5/1	0.9/1	7.2/3	3.8/2	5.9/1	10.9/3	7.4/3	0.7/2	8.2/2	3.8/3
*Lactobacillus helveticus*	0	0.2	0.16	0.04	0.0	0.1	0.09	0.02	0.17	0.2
*Lactobacillus parabuchneri*	0.2/1	0	0.8/1	0.09/1	0.2/1	2.1/1	0.1/1	0.02/1	1.2/1	0.02/1
*Lactobacillus parafarraginis*	0	0	0.2	0.02	0	0.3	0.06	0.05	0.2	0
*Lactobacillus plantarum*	9.6	0.2	0.2	0.2	0	0.09	0.06	0	0	0
*Lactobacillus rhamnosus*	0	0.04	0.1	0.4	0.6	0.2	0.6	0	0.2	0.4
	**Pecorino Siciliano**^**†**^									
*Lactobacillus* sp.	18.6/2	56.7/1	31.2/2	53.8/2	58.8/2	10.4/3	55.9/2	51.3/1	27.2/2	53.9/1
*Peptostreptococcus stomatis*	0.2	0	0	0	0	0	0	0	0	0
*Planococcus* sp.	0.6	0	0	0	0	0	0	0	0	0
*Staphylococcus* sp.	0.3/1	0.04/1	0	0	0.03/1	0	0	1.06/1	0	0
*Staphylococcus equorum*	0.8/1	1.6/1	0.3/1	0.3/1	0.2/1	0.2/1	0.4/1	1.3/1	0.1/1	2.0/1
*Streptococcus lutetiensis*	0	0.04/1	0.06/1	0.1/2	0.1/1	0.1/1	0.2/2	0.17/2	0.1/2	0
*Streptococcus macedonicus*	3.5/2	13.0/2	9.1/2	6.3/2	5.2/2	0.6/2	2.2/2	29.4/4	14.6/2	17/2
*Streptococcus* sp.	2.4/1	0.6/1	1.5/1	3.1/1	3.3/2	0.65/1	1. 2/1	2.1/1	1.5/1	2.8/1
*Streptococcus thermophilus*	45.7/2	25.1/2	48.3/3	29.3/2	24/3	72.3/2	31.1/2	15.4/2	44.5/3	18.9/2
*Tetragenococcus halophilus*	0	0.8	0.04	0.02	0.01	0	0.06	0.16	0.16	0.16
	**Pecorino Toscano**									
*Brevibacterium* sp.	0.7/5	0.9/4	4.4/4	3.1/4	0.2/4	0.09/2	0.8/3	0.2/2	0.1/2	0.6/4
*Brachybacterium sp*	0.5	0.3	0.9	0.6	0.09	0	0.01	0.07	0.02	0.09
*Flavobacterium* sp.	0	0.02	0.3	0	0	0	0	0	0	0
*Halomonas variabilis*	0	0.7/3	2.6/2	0.2/2	0.09/2	0	0	0.07/2	0	0.04/1
*Lactococcus garvieae*	0	0	0.06	0.1	0	0	0	0	0	0.02
*Lactococcus lactis*	98.1/5	97.5/5	90.9/6	95.6/6	99.4/5	99.9/7	98.8/5	99.5/5	99.4/6	98.5/7
*Psychrobacter* sp.	0	0	0	0	0.1	0	0.04	0	0	0
*Staphylococcus equorum*	0.3	0.3	0.3	0.4	0.06	0	0.1	0.1	0.5	0.6
*Staphylococcus* sp.	0	0.1	0.02	0	0	0	0	0	0	0.02
*Streptomyces* sp.	0	0	0.3	0	0	0	0	0	0	0

Number and abundance of OTUs assigned to the *Actinobacteria*, *Bacteroidetes*, *Firmicutes*, *Proteobacteria* species level, when such assignment was possible, of Fiore Sardo, Pecorino Siciliano and Pecorino Toscano cheeses. Only OTUs occurring at 0.1% abundance in at least one sample were included. Based on 16S rRNA gene pyrosequencing analysis of all RNA samples directly from Fiore Sardo, Pecorino Siciliano and Pecorino Toscano cheeses.

^**‡**^Number of OTUs with the same assignment refers to OTU that have been combined into a single entry, detected either within the same cheese or in different cheeses. Cells wherein the number of OTU is not reported implies that only one OTU was assigned.

^§^Slice of each cheese was cut into nine sub-blocks identified by the letters A—I. Sub-blocks A, D, and G, and sub-blocks C, F and I were collected from top and bottom surface region, respectively, whereas sub-blocks B and H from inner side region, and sub-block E from the core. The whole slice was the control. Further details were reported in the Material and Methods and in Fig 1.

^**†**^The total number of OTUs for FS, PS and PT was 29, 51 and 29, respectively.

### Correlations among microbiota, proteolysis and volatile components

**Fig 6:**
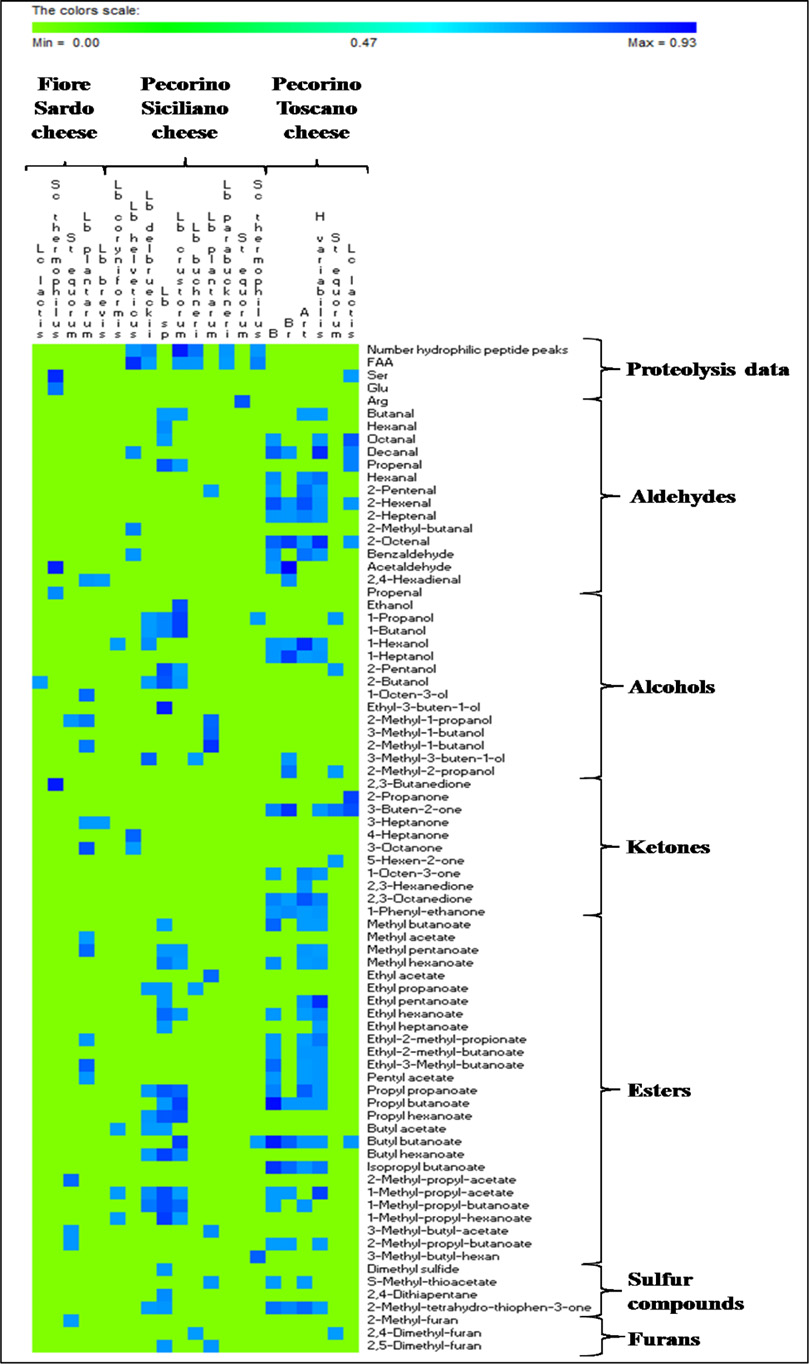
Correlations between the abundance of operational taxonomic units (OTUs) and proteolysis and synthesis of volatile components. Correlations between the abundance of OTUs and total free amino acid (FFA), concentration of Ser, Glu, Asp, and Arg, number of hydrophilic peaks of the pH 4.6-soluble nitrogen fractions, and volatile components (arbitrary units of area) identified from Fiore Sardo, Pecorino Siciliano, and Pecorino Toscano cheeses. Euclidean distance and McQuitty’s criterion (weighted pair group method with averages) were used for clustering. The colors correspond to normalized mean data levels from low (grey) to high (yellow). The color scale, in terms of units of standard deviation, is shown at the top. Only the positive correlations with a P<0.05, FDR<0.05 and r>0.7 are reported. *Lactococcus lactis*, Lc. lactis; *Streptococcus thermophilus*, Sc. thermophilus; *Staphylococcus equorum*, St. equorum; *Lactobacillus plantarum*, Lb. plantarum; *Lactobacillus brevis*, Lb. brevis; *Lactobacillus coryniformis*, Lb. coryniformis; *Lactobacillus helveticus*, Lb. helveticus; *Lactobacillus delbrueckii*, Lb. delbruekii; *Lactobacillus* sp., Lb. sp; *Lactobacillus crustorum*, Lb. crustorum; *Lactobacillus buchneri*, Lb. buchneri; *Lactobacillus parabuchneri*, Lb. parabuchneri; *Brevibacterium* sp., B; *Brachybacterium* sp., Br; *Halomonas variabilis*, H. variabilis; Art, *Arthrobacter*.

All the 87 OTUs were used to find correlations. Only positive correlations (p<0.05; FDR<0.05; r>0.7) were further detailed (Fig 6). Regarding FS cheese, the low abundance of *L*. *plantarum* was positively correlated with aldehydes (2,4-hexadienal and benzaldehyde), and esters (methyl and branched esters). *L*. *brevis* was positively correlated with 2,4-hexadienal and 3-heptanone, and *Sc*. *thermophilus* with acetaldehyde and propenal, 2,3-butanedione and Ser and Glu. The concentration of total FAA and number of hydrophilic peptide peaks of PS cheese correlated (p<0.05; r>0.6) with the abundance of *L*. *delbruecki*, *Sc*. *thermophilus*, *L*. *helveticus*, *L*. *buchneri*, *L*. *crustorum* and *L*. *parabuchneri*. Regarding VOC, the abundance of *Lactobacillus* sp. was positively correlated with aldehydes (n-alkanals, branched), some primary and secondary alcohols, many esters (methyl, ethyl, propyl, butyl, 1-methyl-propyl), and sulfur compounds (e.g., dimethyl sulfide). Although matching with different compounds, *L plantarum* and *L*. *crustorum* were correlated with the same chemical classes. *L*. *helveticus* was correlated with some aldehydes and ketones, and *L*. *delbruecki* with some esters and alcohols. The correlations found for PT cheese regarded the abundance of *Brevibacterium* sp. with a very large number of VOC (14 esters, 9 aldehydes, 2 alcohols, 4 ketones, and 2 sulfur compounds). *Brachybacterium* sp., *Arthrobacter* and *H*. *variabilis* also correlated with several aldehydes (e.g., 2-heptenal), alcohols (e.g., 1-hexanol), esters (e.g., propyl butanoate), ketones (e.g., 2,3-octanedione) and sulfur compounds (2-methyl-tetrahydro-thiophen-3-one). *Lc*. *lactis* correlated with the concentration of Ser.

## Discussion

Bacterial growth on solid matrices (e.g., cheese) is colonial. Interactions between colonies occur, which affect both the spatial distribution of bacteria and the consequent localization of bacterial enzymes into the cheese matrix [10]. This study aimed at explaining how the microbiota is spatially distributed in three Italian PDO ewes’ milk cheeses (chosen as model systems), and how this drives the maturation (proteolysis and synthesis of volatile compounds) of the cheeses.

The three Italian ewes’ milk cheeses were chosen since they are manufactured using various technology options and have the recognition of PDO. As such, continuous insights are desirable to underline and explain their typical features. This should ensure a high quality of production, which is also affect through the spatial distribution of the microbiota. FS and PS cheeses are manufactured from raw ewes’ milk, without starter cultures. The moulded curd of PS cheese is heated in hot whey at 80°C for ca. 20 min. PT cheese is manufactured with pasteurized milk and selected autochthonous cultures of *Lactococcus lactis*.

Mean values for the gross composition, as routinely determined by analyzing the entire cheese slice, approached those previously found for these varieties [25, 43–44]. The gradients found for moisture, and concentrations of salt, fat and protein were confirmatory. The values of pH differed only within sub-blocks of PT cheese. The pH, salt and a_*w*_ were, therefore, some of the factors that varied between cheese sub-blocks [7, 8].

FS and PT harbored the highest cell density of mesophilic cocci, while mesophilic lactobacilli were the most abundant in PS cheese. PT cheese, manufactured with pasteurized milk, contained the lowest value of adventitious mesophilic lactobacilli [45]. In term of cell density of presumptive microbial groups, the abundance did not follow a specific spatial distribution. The only exception was PS cheese. The lowest number of mesophilic lactobacilli was found in the core E and adjacent D and F sub-blocks. After heating the moulded curd in hot whey, these cheese regions are subjected to a slower cooling and to a prolonged exposition to high temperature.

The general pattern of proteolysis of many cheese varieties may be summarized as a sequential primary (by residual coagulant activity and/or by plasmin and proteinases and peptidases of primary starters) and secondary (by NSLAB and secondary starters) proteolysis occurring throughout cheese ripening [13]. After ripening, the primary proteolysis did not differ between cheese sub-blocks, the only differences regarded the varieties. FS and PT cheeses showed the complete hydrolysis of α_s_1-CN. Unlike other Italian ewes’ milk cheeses (e.g., Pecorino Romano and PS cheeses), the manufacture of FS and PT cheeses does not include cooking of the curd, which allows a persistent chymosin activity [26, 46]. As the primary proteolysis was observed only at the end of ripening, a spatial variability at cheese sub-blocks level during the early ripening could be not excluded. Secondary proteolysis differed between sub-blocks of each cheese, especially when the number and area of hydrophilic and hydrophobic peptide peaks were analyzed. Sub-blocks containing the highest number and area of peptide peaks varied among the cheeses (e.g., sub-block D, E and F for PS, and C, B, I and F for PT cheese), which agreed with the concentration of FAA.

Secondary proteolysis resulting into shorter peptides and free amino acids might be related to *L*. *delbrueckii and St*. *thermophilus*, the most abundant species identified in sub-blocks D, E and F of PS cheese, and to *Lc*. *lactis* found in sub-blocks C, B, I and F of PT cheese. The proteolytic system of these species was in depth investigated, and a model for casein breakdown, transport, degradation of casein-derived peptides, and regulation was widely established [47]. Secondary proteolysis in sub-blocks of FS cheese was almost uniform. Probably, this reflected the uniform distribution of the most abundant species. The sub-block D of FS cheese showed the lowest concentration of peptides, whereas the other sub-blocks occupied an almost uniform and separated zone of the plane. Although these differences, it seemed that all the cheese regions contained enough precursors (e.g., FAA) for synthesizing VOC.

The levels of most VOC varied between sub-blocks of each cheese. Regardless of the cheese variety, the poorest profile of VOC was restricted to the core E region. The lowest oxygen availability of this region might have decreased the synthesis of VOC, directly for compounds generated via oxidation (e.g., oxidative synthesis of aldehydes) [48], and indirectly because of the selective pressure on bacterial diversity (see below). As the consequence of the low redox potential of the core, the sub-block E showed high levels of alcohols, which are synthesized from the reduction of aldehydes (primary alcohols such as methanol, ethanol, 1-propanol, 1-butanol, 1-hexanol in FS and PS cheese) and methyl-ketones (secondary alcohols such as 2-propanol, 2-butanol, 2-pentanol in PS cheese). The corresponding esters (methyl, ethyl, propyl, butyl, 1-methyl-propyl) were synthesized at high levels from the above alcohols. The VOC composition differed between under rind sub-blocks of FS cheese. A, B and F sub-blocks the richest in aldehydes, diones and sulfur compounds were opposed to the top (D and G) and bottom (C and I) sub-blocks, which were characterized for their richness for almost all the other compounds. Such differences were hence related to a global level of activity, not to a particular metabolism. Only block H may show a more oxidized state as determined by the ratio aldehydes/alcohols. Overall, ketones were the largest class of VOC in FS cheese. 2,3-Butanedione is synthesized from pyruvate, lactose or citrate by lactic acid bacteria, especially by *Lc*. *lactis*, which was found to be the most abundant species for this cheese [49]. The VOC distribution of PS cheese varied between sub-blocks. Presumably, these differences were related to metabolisms determined by the various spatial distributions of bacteria. The top (A, D and G) and inner side (H) sub-blocks of PS were rich in compounds coming from amino-acid catabolism (branched and sulfur compounds) and in aldehydes synthesized via oxidation. The bottom sub-blocks C, I and F were rich in ketones coming from microbial oxidation. Heating of PS curd has selected a thermophilic microbiota, mainly consisting of lactobacilli. These bacteria may synthesize alcohols and related esters both under reduced (core E) or less oxygenated (rind block F) conditions. This agrees with the correlation which was found between many lactobacilli and these VOC. The presence of ketones may be related to the eventual growth of a surface microbiota, mainly at early stage of ripening, which was not investigated in this study. The VOC profile of PT cheese differentiated mainly the top under rind sub-blocks A, D and G (the richest in esters, ketones and branched alcohols, and poor in aldehydes) and the side-bottom blocks H and I (the richest in aldehydes and sulfur compounds). Aldehydes are unstable compounds that are reduced to alcohols or oxidized to acids, indicating the progressive cheese maturation [50, 51]. The identified *Brevibacterium*, *Brachybacterium*, *Arthrobacter* and *H*. *variabilis*, together with yeasts and moulds, which were not investigated in this study, were certainly responsible for the synthesis of the above compounds. They exert a strong activity of amino-acid catabolism and synthesis of carbonyls (aldehydes, ketones) and esters [52]. Regardless of the cheese variety, biotic (see below) factors determined the VOC richness of under rind sub-blocks. Discontinuous turning of the cheese curd during ripening might also have affected the differences between top and bottom under rind sub-blocks of PS and PT cheeses.

The total 87 OTUs (29, 51 and 29 for FS, PS and PT respectively) represented active members of the microbial community. Top and bottom under rind sub-blocks of all three cheeses contained the widest biodiversity (15–24 and 10–27 OTUs, respectively) [11, 12]. The largest diversity that was found in the control (whole cheese slice) mainly referred to OTUs (15–31) with very low abundance, which could be undetectable using individual cheese sub-blocks. Regardless of the sub-block, *Lc*. *lactis* was the most abundant species found for FS cheese, followed by *Sc*. *thermophilus* and *Lactobacillus* sp. Previous studies on Fiore Sardo and other ewes’ milk cheeses, also made without starters, confirmed the abundance of *Lc*. *lactis* [27, 53–55]. The microbiota diversity that of these other cheeses was higher diversity than that found under the experimental conditions of this study. This suggested that the metabolically active fraction of bacteria consisted only of a few highly abundant (or active) species. The distribution of mesophilic lactobacilli was dispersed in the space. The core E (19 OTUs) and adjacent D and F under rind sub-blocks (18 and 19 OTUs, respectively) harbored *Lactobacillus parabuchneri*. *Lactobacillus* sp. was manly found in the top under rind (A, D and G) (19, 18 and 15 OTUs, respectively), and *Lactobacillus brevis* throughout under rind. The bacterial structure and the differential spatial distribution of PS cheese was the most complex. *Sc*. *thermophilus*, followed by *Lactobacillus delbrueckii* and *Lactobacillus helveticus*, were the most abundant species found in the core E (23 OTUs), and adjacent top D and bottom F (24 and 27 OTUs, respectively) under rind sub-blocks. At a considerable abundance (51.3–58.8%), *Lactobacillus* sp. was distributed around, in the other sub-blocks. Probably, this was determined by the lower resistance of mesophilic lactobacilli to the persistent high temperature in the cheese core region [11]. Besides this clearly separated spatial distribution, an almost dispersal localization of several species (*Lactobacillus coryniformis*, *L*. *crustorum*, *L*. *parabuchneri*, *L*. *buchneri*, *L*. *rhamnosus*, *L*. *plantarum* and *L*. *parafarraginis*) was found. The under rind and inner side (B and H) sub-block microbiota (21 and 24 OTUs, respectively) also contained *Streptococcus macedonicus*, *Staphylococcus equorum*, *Tetragenococcus halophilus* and *Enterococcus faecium*. The environment that characterized these cheese regions favored the colonization/persistence of aerobic, aero-tolerant or halophilic bacteria, being halophilic species also inoculated via brining process [9, 11, 12]. PT was the cheese with the lowest biodiversity (15 OTUs for the control). The starter *Lc*. *lactis* was the most abundant species, and the only one that was found in the cheese core (9 OTUs). According to previous studies [12, 55], top under rind sub-blocks (A, D and G) (16, 20 and 18 OTUs, respectively) harbored *Brevibacterium* sp., *Brachybacterium* sp. and *Halomonas variabilis*.

As showed by the positive correlation analysis, the differential spatial distribution of the microbiota within cheeses statistically (p<0.05; r>0.7) agreed with the VOC distribution or FAA concentration. Despite their low relative abundance, mesophilic lactobacilli (mainly *L*. *plantarum*) were the only microbial group positively correlated with several VOC, like esters, alcohols, aldehydes and sulfur compounds. These adventitious mesophilic lactobacilli are a significant proportion of the microbial population of many ripened cheese varieties, being also used as adjunct cultures [13, 56]. The abundance of the thermophilic lactic acid bacteria found in PS cheese was positively correlated with the total concentration of FAA. Although the role of aerobic, aero-tolerant or halophilic bacteria in cheese ripening is still unclear, their abundance was correlated with some FAA and several VOC (e.g., ketones, esters, sulfur compounds, aldehydes and furans). In particular, the synthesis of volatile sulfur compounds in cheese during ripening was almost exclusively attributed to surface bacteria, mainly *Brevibacterium linens* [57]. Overall, sulfur compounds result from the bioconversion of L-methionine to methanethiol through demethiolating activity. Methanethiol is the direct precursor of diverse sulfur aroma compounds such as dimethyl disulfide, dimethyl trisulfide, methional and S-methyl-thioesters [58, 59].

Owing to the large size and the prolonged ripening, hard-cheeses could be affected by a differential spatial distribution of the microbiota. This is the case of the three Italian PDO ewes’ milk cheeses chosen as model systems, even with differences among the varieties. Although, this study is a picture of three Italian PDO ewes’ milk cheeses after four months of ripening, clear differences emerged between cheese core and the other regions, in terms of microbial diversity and correlated synthesis of VOC. Top and/or bottom under rind sub-blocks of all three cheeses contained the significantly widest biodiversity. This diversity was rather low but statistically correlated with secondary proteolysis events and/or synthesis of VOC.

## Supporting Information

S1 Fig Reverse-Phase High Protein Liquid Chromatography (RP-HPLC) of the pH 4.6-soluble nitrogen fraction of Fiore Sardo, Pecorino Siciliano and Pecorino Toscano cheeses. RP-HPLC of the pH 4.6-soluble nitrogen fraction of Fiore Sardo (A), Pecorino Siciliano (B) and Pecorino Toscano (C). Arrows refer to hydrophilic and hydrophobic peptide peak. Slice of each cheese was cut into nine sub-blocks identified by the letters A—I. Sub-blocks A, D, and G, and sub-blocks C, F and I were collected from top and bottom surface region, respectively, whereas sub-blocks B and H from inner side region, and sub-block E from the core. The whole slice was the control. Further details were reported in the Material and Methods and in Fig 1.
(TIF)

S2 Fig Principle Coordinate Analysis (PCoA) based on unweighted UniFrac analysis of all RNA samples of Fiore Sardo (A), Pecorino Siciliano (B), and Pecorino Toscano (C) cheeses.
(TIF)

S3 Fig Principle Coordinate Analysis (PCoA) based on weighted UniFrac analysis of all RNA samples of Fiore Sardo (A), Pecorino Siciliano (B), and Pecorino Toscano (C) cheeses.
(TIF)

S1 Table Main chemical composition^a^ of Fiore Sardo, Pecorino Siciliano and Pecorino Toscano cheeses. (PDF)

S2 Table Free amino acids (FAA) found in Fiore Sardo, Pecorino Siciliano, and Pecorino Toscano cheeses. Mean values^a^ for the level of FAA (mg/kg) found in Fiore Sardo, Pecorino Siciliano, and Pecorino Toscano.
(PDF)

S3 Table Volatile components (VOC) identified in Fiore Sardo, Pecorino Siciliano, and Pecorino Toscano cheeses. Concentrations of VOC (log of units of area) identified in Fiore Sardo, Pecorino Siciliano and Pecorino Toscano.
(PDF)

S4 Table Observed diversity and estimated sample coverage of 16S rRNA sequencing analysis of Fiore Sardo, Pecorino Siciliano and Pecorino Toscano cheeses. (PDF)
