# Supplementary figures and images for: Spatial Distribution of the Metabolically Active Microbiota within Italian PDO Ewes' Milk Cheeses
Source: PLoS One. 2016 Apr 13;11(4):e0153213. doi: 10.1371/journal.pone.0153213 (PMC4830609; doi:10.1371/journal.pone.0153213)

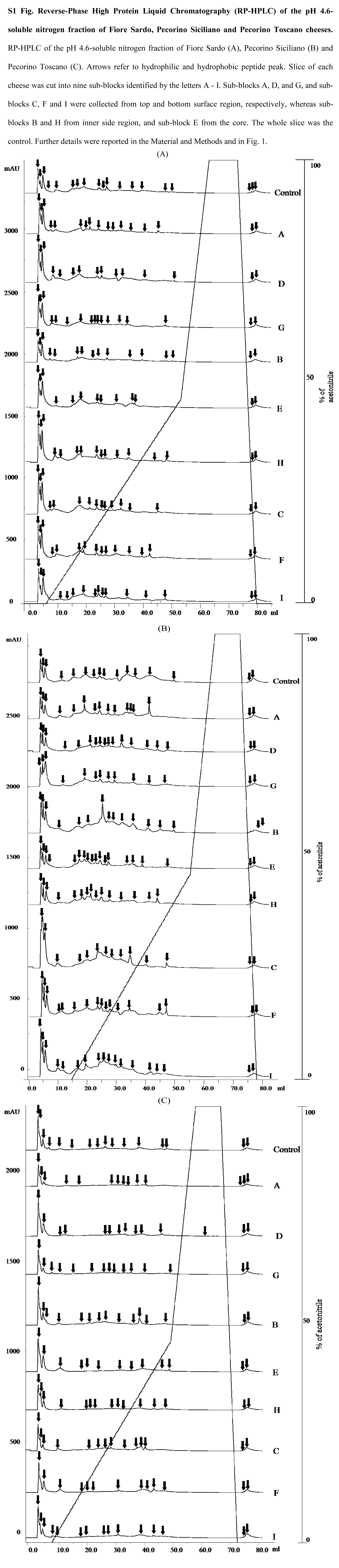

Supplement: S1 Fig — RP-HPLC of the pH 4.6-soluble nitrogen fraction of Fiore Sardo (A), Pecorino Siciliano (B) and Pecorino Toscano (C). Arrows refer to hydrophilic and hydrophobic peptide peak. Slice of each cheese was cut into nine sub-blocks identified by the letters A—I. Sub-blocks A, D, and G, and sub-blocks C, F and I were collected from top and bottom surface region, respectively, whereas sub-blocks B and H from inner side region, and sub-block E from the core. The whole slice was the control. Further details were reported in the Material and Methods and in Fig 1. (TIF) [file pone.0153213.s001.tif]

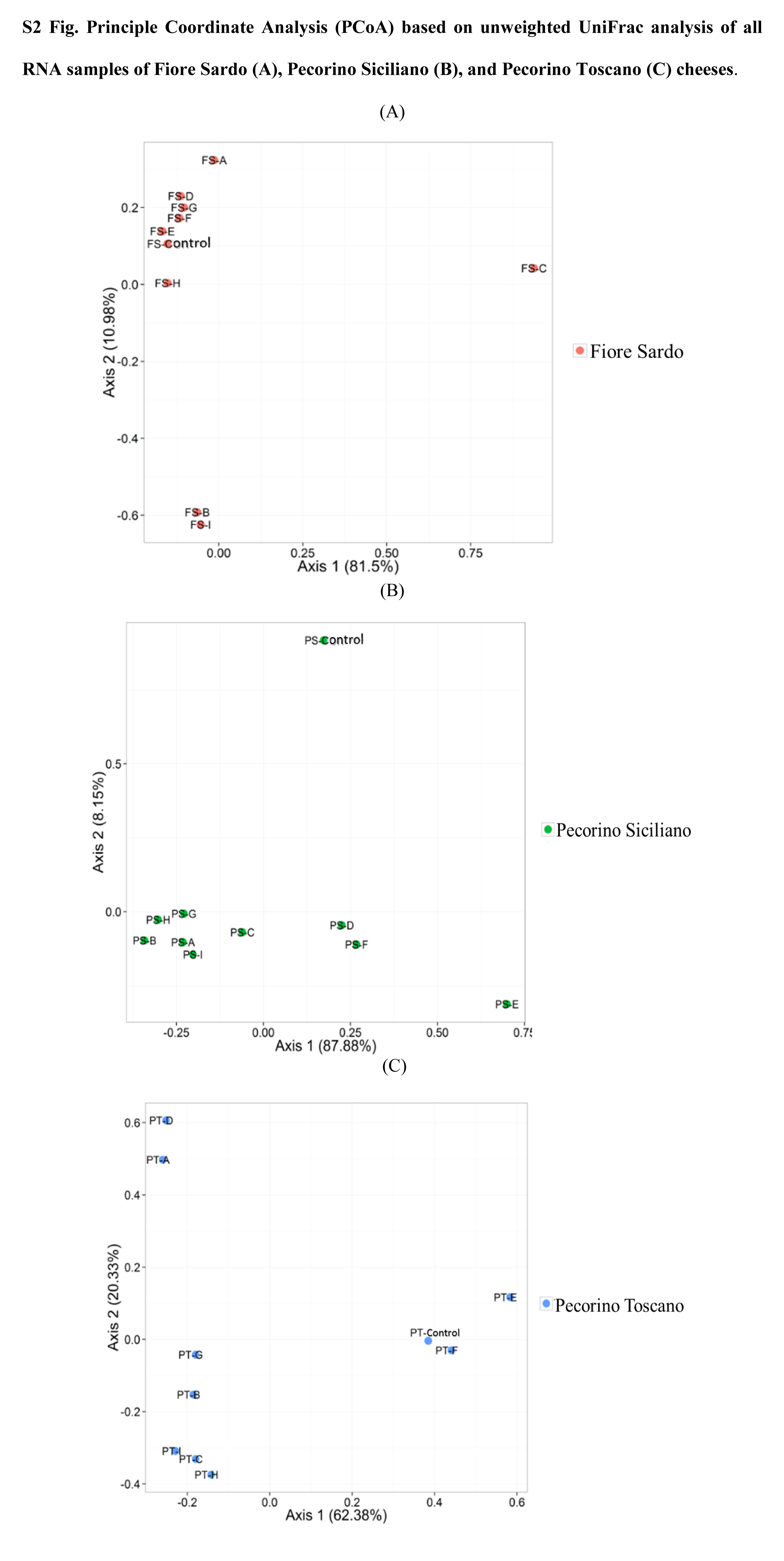

Supplement: S2 Fig — Principle Coordinate Analysis (PCoA) based on unweighted UniFrac analysis of all RNA samples of Fiore Sardo (A), Pecorino Siciliano (B), and Pecorino Toscano (C) cheeses. (TIF) [file pone.0153213.s002.tif]

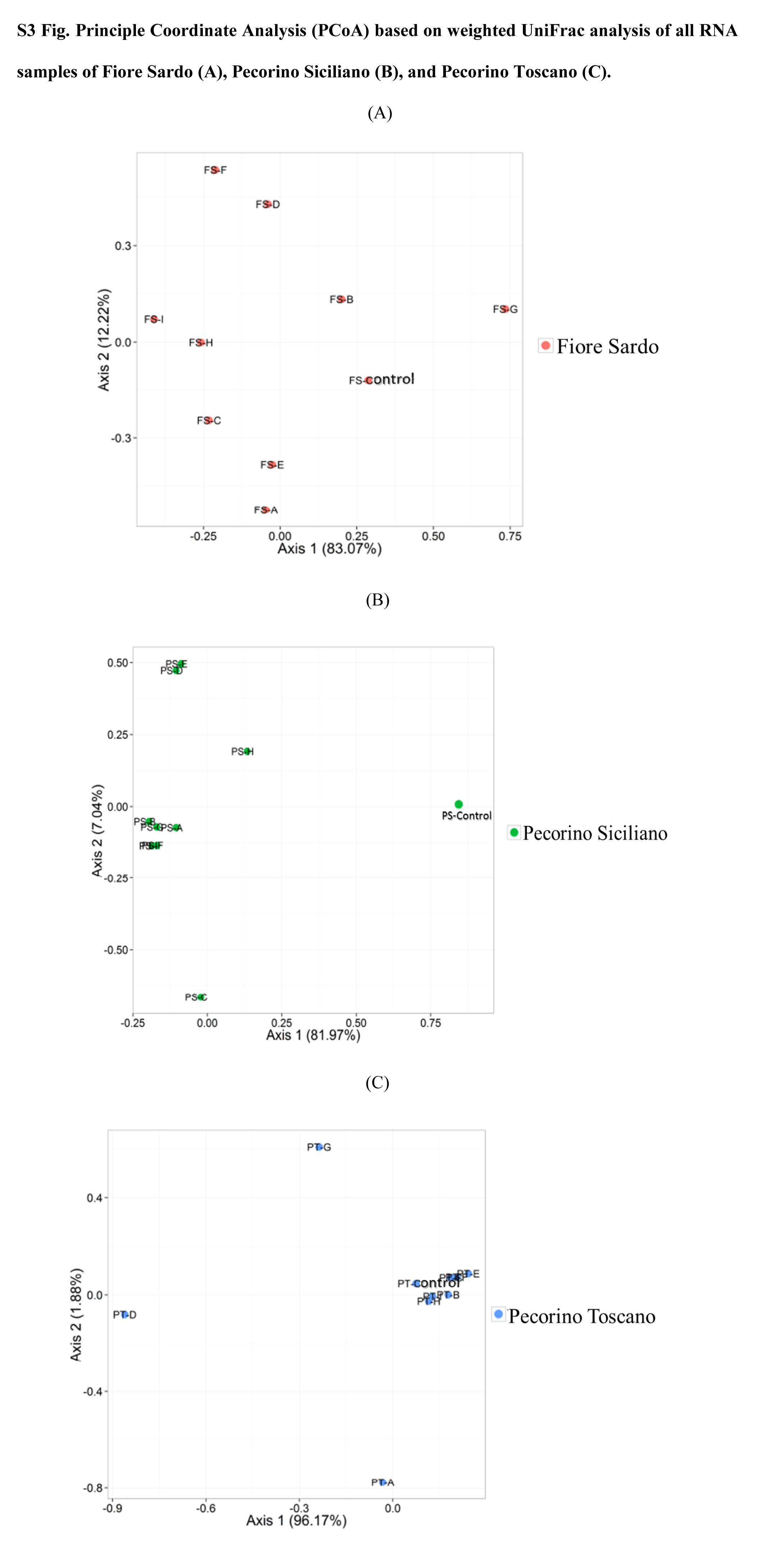

Supplement: S3 Fig — Principle Coordinate Analysis (PCoA) based on weighted UniFrac analysis of all RNA samples of Fiore Sardo (A), Pecorino Siciliano (B), and Pecorino Toscano (C) cheeses. (TIF) [file pone.0153213.s003.tif]
