# Supplementary material for: Spatial Distribution of the Metabolically Active Microbiota within Italian PDO Ewes' Milk Cheeses
Source: PLoS One. 2016 Apr 13;11(4):e0153213. doi: 10.1371/journal.pone.0153213 (PMC4830609; doi:10.1371/journal.pone.0153213)
Supplement: S1 Table — (PDF) [file pone.0153213.s004.pdf]

S1 Table. Main chemical composition[*] of Fiore Sardo, Pecorino Siciliano and Pecorino Toscano cheeses.

	Control[§]	A	D	G	B	E	H	C	F	I
	**Fiore Sardo**									
Mean pH ± SD	5.78±0.4^{d}	5.83±0.3^{b}	5.85±0.4^{a}	5.80±0.2^{c}	5.81±0.2^{c}	5.80±0.1^{c}	5.81±0.3^{b}	5.80±0.1^{c}	5.83±0.2^{b}	5.82±0.1^{c}
Mean moisture content (%) ± SD	19.2±1.4^{b-c}	12.1±1.2^{g}	18.2±1.2^{c}	12.0 ±1.1^{f}	12.5±1.3^{f}	25.2±1.3^{a}	12.0±1.3^{f}	13.0±1.2^{e}	19.2±1.1^{b}	13.4 ± 1.4^{d}
Mean fat content (%) ± SD	27.5±1.6^{b}	28.2±1.3^{a}	26.5 ±1.1^{c}	27.8 ±1.4^{b}	28.1±1.2^{a}	25.1±1.0^{d}	28.5±1.2^{a}	27.9±1.4^{b}	27. ±1.2^{b-C}	28.3±1.3^{a}
Mean protein content (%) ± SD	26.1±1.4^{c}	26.9±1.1^{b}	25.1±1.1^{d}	27.0±1.2^{a}	27.2±1.0^{a}	24.4±1.3^{e}	26.7±1.3^{b}	26.8±1.2^{b}	25.0±1.2^{d}	27.1±1.2^{a}
Mean NaCl content (%) ± SD	4.2±0.04^{d}	4.5±0.01^{b}	4.0±0.04^{d}	4.6±0.01^{a}	4.4±0.02^{c}	4.1±0.05^{e}	4.5±0.02^{b}	4.6±0.03^{a}	3.9±0.04^{f}	4.4±0.02^{c}
	**Pecorino Siciliano**									
Mean pH ± SD	5.73±0. 4^{c-d}	5.72±0.1^{d}	5.74±0.2^{c-d}	5.70±0.2^{d}	5.71±0.2^{d}	5.9±0.3^{a}	5.73±0.2^{c-D}	5.81±0.4^{b}	5.75±0.2^{c}	5.79±0.3^{c}
Mean moisture content (%) ± SD	18.8±1.3^{d}	15.2±1.0^{e}	22.0±1.2^{c}	15.5±1.4^{e}	14.6±1.2^{f}	25.0±1.1^{a}	15.3±1.2d^{e}	14.7±1.3^{f}	23.0±0.9^{b}	14.9±1.1^{f}
Mean fat content (%) ± SD	18.4±1.4^{c}	19. ±0.9^{a-B}	18.7±1.2^{c}	19.0±0.9^{b}	19.7±1.1^{a}	17.3±1.1^{d}	19.1±0.7^{b}	19.9±1.2^{a}	17.1±1.4^{d}	19.4±1.4^{a-b}

Mean protein content (%) ± SD	30.6±1.9^{cd}	31.5±1.8^{b}	28.9±1.0^{ef}	31.4±1.1^{b}	30.4±1.2^{d}	28.1±1.7^{f}	31.1±1.2^{c}	30.2 ±1.1^{d}	29.6±1.2^{e}	31.9±1.3^{a}
Mean NaCl content (%) ± SD	3.8±0.02^{e}	4.3±0.01^{c}	3.6±0.04^{f}	3.9±0.01^{d}	3.9±0.03^{d}	3.5±0.05^{f}	4.5±0.03^{a}	4.3±0.02^{c}	3.3±0.02^{g}	4.4±0.01^{b}
	**Pecorino Toscano**									
Mean pH ± SD	5.81±0.3^{b}	6.01±0.2^{a}	6.03±0.1^{a}	6.04±0.2^{a}	5.6±0.1^{d}	5.51±0.3^{e}	5.7±0.2^{c}	5.8±0.2^{b}	5.71±0.2^{c}	5.6±0.2^{d}
Mean moisture content (%) ± SD	18.5±1.4^{b-c}	13.1±1.2^{d-e}	18.0±1.3^{b}	12.1±11^{e}	12.5±1.1^{e}	20±1.2^{a}	13.7±1.3^{d}	13.4±1.2^{d-E}	17.0±1.0^{c}	13.4±1.2^{d}
Mean fat content (%) ± SD	19.2±1.8^{c}	20.1±1.4^{b}	18.4±1.3^{d}	20.4±1.4^{b}	20.4±1.4^{b}	17.9±1.4^{e}	21.0±1.5^{a}	20.0 ± 1.2^{b}	18.6±1.5^{d}	20.4±1.0^{b}
Mean protein content (%) ± SD	28.4±1.7^{d}	29.1±1.0^{c}	28.9±1.1^{c}	29.4±1.2^{b}	29.7±0.9^{a}	28.4±1.0^{d}	29.3±0.9^{b}	29.0±1.2^{c}	28.7±1.3^{d}	29.6±0.8^{a}
Mean NaCl content (%) ± SD	3.2±0.07$_{h}$	4.0±0.02^{d}	3.8±0.02^{e}	4.3±0.02^{d}	4.1±0.03^{c}	3.4±0.03^{g}	4.4±0.02^{a}	4.2±0.01^{b}	3.7±0.04^{f}	4.1±0.01^{c}

Data in the same column with different superscript letters (a-h) are significantly different ($P<0.05$).

*Mean values ± standard deviations for three batches of each type of cheese, analysed in duplicate.

[§]Slice of each cheese was cut into nine sub-blocks identified by the letters A - I. Sub-blocks A, D, and G, and sub-blocks C, F and I were collected from top and bottom surface region, respectively, whereas sub-blocks B and H from inner side region, and sub-block E from the core. The whole slice was the control. Further details were reported in the Material and Methods and in Fig. 1.
