# Supplementary material for: Spatial Distribution of the Metabolically Active Microbiota within Italian PDO Ewes' Milk Cheeses
Source: PLoS One. 2016 Apr 13;11(4):e0153213. doi: 10.1371/journal.pone.0153213 (PMC4830609; doi:10.1371/journal.pone.0153213)
Supplement: S2 Table — Mean valuesa for the level of FAA (mg/kg) found in Fiore Sardo, Pecorino Siciliano, and Pecorino Toscano. (PDF) [file pone.0153213.s005.pdf]

S2 Table. Free amino acids (FAA) found in Fiore Sardo, Pecorino Siciliano, and Pecorino Toscano cheeses. Mean values* for the level of FAA (mg/kg) found in Fiore Sardo, Pecorino Siciliano, and Pecorino Toscano.

	**Mean concentration of FAA (mg/kg)**									
**FAA**	**Control***	**A**	**D**	**G**	**B**	**E**	**H**	**C**	**F**	**I**
	**Fiore Sardo**									
Asp	458.4	423.5	313.7	410.5	399.2	337.5	484.4	481.4	410.5	364.8
Thr	225.4	285.7	151.0	196.5	189.8	151.7	231.9	340.0	196.5	181.6
Ser	1182.7	814.3	514.4	842.2	1009.7	1027.1	875.8	560.8	842.2	856.6
Glu	1362.4	1304.2	1151.7	1331.8	1246.7	1175.9	1343.0	1382.9	1331.8	1242.6
Gly	177.5	157.1	127.8	157.0	152.2	130.9	180.6	441.1	157.0	150.2
Ala	531.9	441.8	492.7	502.5	472.5	466.3	528.7	388.6	502.5	476.7
Cys	59.8	106.0	60.4	0.0	0.0	58.2	1109.1	835.5	0.0	64.0
Val	1003.4	831.9	814.3	994.3	949.0	946.6	673.8	1011.4	994.3	950.9
Met	479.2	512.4	467.8	463.5	416.7	414.1	595.1	534.7	463.5	395.1
Ile	581.8	612.4	482.8	572.3	507.9	464.2	953.4	646.0	572.3	479.0
Leu	1329.9	1155.3	912.7	1347.1	1267.2	1270.3	960.9	1386.2	1347.1	1236.4
Tyr	551.7	455.6	533.2	703.2	616.4	514.2	1066.8	743.7	703.2	380.5
Phe	1377.2	1116.0	1391.8	1368.5	1222.6	1278.8	1157.0	1465.4	1368.5	1258.5
His	668.7	535.0	53.3	677.0	615.6	636.5	281.8	689.6	677.0	643.3
Trp	107.2	74.5	204.7	175.1	74.0	207.7	95.3	255.3	175.1	151.0
Orn	474.5	135.5	401.7	457.5	173.5	472.7	125.4	404.0	457.5	272.6
Lys	835.2	738.4	1020.6	807.2	758.5	801.3	784.4	850.0	807.2	814.0
Arg	1101.9	1203.2	71.1	1068.8	1010.8	997.3	1098.3	1137.0	1068.8	1033.1
Pro	82.0	60.5	812.3	79.8	75.0	68.7	92.1	97.2	79.8	41.2
**Total**	12590.8 ± 72.5^{a}	10936.5 ± 44.5^{b}	9977.9 ± 32.4^{c}	12637.7 ± 31.5a	11157.3 ± 58.4^{b}	11420.1 ± 42.1^{b}	12201.7 ± 79.1^{a}	12540.5 ± 75.5^{a}	12155.0 ± 48.5^{a}	10992 ± 38.50^{b}
	**Pecorino Siciliano**									
Asp	973.4	655.2	1283.2	568.9	729.4	1461.5	76.3	432.3	1084.4	497.4
Thr	452.6	314.0	595.3	267.8	406.5	625.5	504.8	232.7	486.3	236.5
Ser	1055.3	772.4	1386.4	723.7	774.6	1210.5	301.9	405.7	1133.3	644.3
Glu	1585.5	1104.7	891.4	1220.1	1506.8	1810.5	435.2	1224.7	759.8	1192.4

Gly	242.0	181.1	316.4	155.6	210.5	316.9	157.6	141.1	258.3	128.7
Ala	627.5	497.9	718.2	421.5	592.8	696.8	398.4	422.3	595.7	357.3
Cys	85.6	52.1	111.2	44.5	71.4	126.5	60.2	51.5	93.5	42.2
Val	774.3	511.4	856.1	539.8	720.5	846.5	599.9	538.3	740.0	486.9
Met	641.6	301.4	761.9	405.2	581.4	737.5	397.2	383.5	605.6	344.8
Ile	865.8	638.3	1033.4	541.5	751.8	996.1	512.6	489.9	864.2	486.9
Leu	1009.8	601.4	1146.6	681.5	921.3	1144.5	877.7	652.6	989.9	610.9
Tyr	388.9	321.9	458.1	227.3	325.5	489.1	326.7	260.1	314.0	184.5
Phe	741.9	307.8	889.4	411.4	667.3	998.2	666.2	380.3	742.5	369.6
His	43.4	32.5	8.9	25.3	63.3	10.9	112.7	39.7	9.4	11.8
Trp	125.2	106.9	137.1	110.0	95.7	34.9	92.7	84.5	69.6	88.2
Orn	164.7	140.8	180.4	144.8	364.4	45.9	156.4	265.1	91.6	116.0
Lys	487.0	114.4	631.0	259.6	355.3	755.2	331.3	198.2	539.9	227.3
Arg	1280.6	1028.1	1403.5	977.6	1211.1	1356.2	853.2	928.0	1234.0	902.2
Pro	243.1	246.4	224.8	235.6	285.5	52.0	117.3	243.0	138.6	180.6
**Total**	11788.2 ± 64.9[b]	7928.7 ± 61.8[c]	13033.2 ± 11.4[a]	7961.7 ± 39.4[c]	10634.9 ± 59.5[b]	13715.0 ± 75.6[a]	6978.4 ± 43.4[c]	7373.9 ± 40.73[d]	10750.7 ± 62.9[b]	7108.5 ± 41.1[c]
	**Pecorino Toscano**									
Asp	335.1	148.5	117.2	126.3	297.2	89.0	223.7	253.6	340.3	307.0
Thr	181.0	104.3	60.5	88.8	146.8	51.5	116.5	276.4	237.6	234.0
Ser	573.6	326.0	187.7	216.3	441.3	333.5	444.5	596.8	695.6	777.8
Glu	1017.3	881.6	555.3	501.7	501.4	523.1	874.9	2061.4	880.7	945.1
Gly	104.4	64.7	47.6	62.7	135.8	46.4	77.6	153.7	119.2	152.4
Ala	290.8	130.9	155.5	175.3	367.7	113.2	200.7	308.5	346.8	407.3
Cys	57.0	242.7	299.4	341.2	435.8	299.2	305.8	445.5	439.4	481.9
Val	490.5	301.8	46.4	64.8	296.8	44.4	127.0	87.4	101.3	131.7
Met	262.6	179.6	141.1	165.6	402.4	97.7	180.2	365.3	281.7	397.4
Ile	357.7	216.0	158.0	208.1	412.8	102.0	235.4	376.1	346.5	422.0
Leu	689.2	601.4	523.1	501.4	509.7	549.3	616.1	821.8	743.4	927.3
Tyr	261.0	370.3	231.2	297.1	770.8	194.5	228.4	529.7	383.2	627.8
Phe	476.5	201.1	102.1	477.6	907.8	403.1	453.3	660.3	594.1	720.3
His	89.7	101.1	117.5	132.2	432.7	173.2	123.7	265.0	199.4	293.7
Trp	180.9	72.7	58.0	72.9	120.5	39.9	59.1	159.4	119.6	126.2

Orn	181.3	182.1	258.1	349.3	578.0	186.2	207.2	700.6	472.5	744.0
Lys	183.5	124.4	89.8	130.7	195.5	116.9	160.5	126.8	143.1	200.2
Arg	688.2	516.2	314.1	414.7	680.0	233.0	503.7	586.3	598.2	576.4
Pro	86.9	20.3	24.0	19.7	82.0	20.6	40.5	55.7	32.4	37.8
**Total**	6507.3 ± 32.1^{c}	4785.8 ± 31.9^{c}	3486.5 ± 23.4^{d}	4346.3 ± 41.6^{c}	7714.5 ± 33.1^{b}	3616.6 ± 50.9^{d}	5178.9 ± 26.1^{c}	8830.1 ± 63.7^{a}	7075.0 ± 63.3^{b}	8510.2 ± 72.8^{a}

Data in the same row with different superscript letters (a-d) are significantly different (P<0.05).

[*]Mean values ± standard deviations for three batches of each type of cheese, analysed in triplicate.

[§]Slice of each cheese was cut into nine sub-blocks identified by the letters A - I. Sub-blocks A, D, and G, and sub-blocks C, F and I were collected from top and bottom surface region, respectively, whereas sub-blocks B and H from inner side region, and sub-block E from the core. The whole slice was the control. Further details were reported in the Material and Methods and in Fig. 1.
