# Supplementary material for: Spatial Distribution of the Metabolically Active Microbiota within Italian PDO Ewes' Milk Cheeses
Source: PLoS One. 2016 Apr 13;11(4):e0153213. doi: 10.1371/journal.pone.0153213 (PMC4830609; doi:10.1371/journal.pone.0153213)
Supplement: S3 Table — Concentrations of VOC (log of units of area) identified in Fiore Sardo, Pecorino Siciliano and Pecorino Toscano. (PDF) [file pone.0153213.s006.pdf]

S3 Table. **Volatile components (VOC) identified in Fiore Sardo, Pecorino Siciliano, and Pecorino Toscano cheeses.** Concentrations of VOC (log of units of area) identified in Fiore Sardo, Pecorino Siciliano and Pecorino Toscano.

	Concentration*									
**Component**	**Control§**	**A**	**D**	**G**	**B**	**E**	**H**	**C**	**F**	**I**
	**Fiore Sardo**									
VOC (log of units of area)										
Aldehydes										
Acetaldehyde	6.1^{c}	6.3^{a}	6.1^{c}	6.2^{b}	6.3^{a}	6.1^{c}	6.1^{c}	6.1^{c}	6.2^{b}	6.2^{b}
Butanal	6.0^{a}	5.8^{c}	5.7^{d}	5.8^{c}	5.9^{b}	5.8^{c}	6.0^{a}	5.7^{d}	5.8^{c}	5.8^{c}
Pentanal	7.1^{b}	6.9^{c}	6.5^{h}	6.5^{h}	6.8^{e}	6.7^{f}	7.2^{a}	6.6^{g}	6.7^{f}	6.8^{e}
Hexanal	6.4^{c}	6.5^{b}	6.4^{c}	6.3^{d}	6.5^{b}	6.5^{b}	6.6^{a}	6.3^{d}	6.5^{b}	6.4^{C}
Heptanal	5.8^{b}	5.8^{b}	5.6^{e}	5.5^{f}	5.7^{c}	5.7^{c}	5.9^{a}	5.6^{d}	5.8^{b}	5.6^{d}
Octanal	5.8^{a}	5.6^{c}	5.4^{e}	5.3^{f}	5.5^{d}	5.5^{d}	5.6^{c}	5.5^{d}	5.7^{b}	5.4^{e}
Nonanal	6.0^{c}	6.2^{a}	5.9^{d}	5.8^{e}	6.1^{b}	5.9^{d}	5.9^{d}	6.0^{c}	6.1^{b}	5.9^{d}
Decanal	4.4^{d}	4.8^{a}	4.4^{d}	4.4^{d}	4.5^{c}	4.2^{e}	4.4^{d}	4.4^{d}	4.6^{b}	4.5^{c}
Propenal	5.5^{d}	5.9^{a}	5.1^{g}	5.3^{f}	5.3^{f}	5.4^{e}	5.6^{c}	5.7^{b}	5.5^{d}	5.7^{b}
2-Pentenal	5.4^{b}	5.4^{b}	5.2^{d}	5.2^{d}	5.3^{c}	5.2^{d}	5.5^{a}	5.2^{d}	5.4^{b}	5.2^{d}
2-Hexenal	5.1^{c}	5.1^{c}	5.0^{d}	5.0^{d}	5.1^{c}	5.0^{d}	5.4^{a}	4.9^{e}	5.2^{b}	5.0^{d}
2,4-Hexadienal	5.4^{c}	5.6^{b}	5.7^{a}	5.7^{a}	5.3^{d}	4.9^{f}	5.4^{c}	4.9^{f}	5.2^{e}	5.2^{e}
2-Heptenal	5.5^{b}	5.4^{c}	5.1^{f}	5.2^{e}	5.4^{c}	5.2^{e}	5.8^{a}	5.1^{f}	5.4^{c}	5.3^{d}
2-Octenal	5.0^{b}	5.0^{b}	4.6^{d}	4.8^{c}	5.1^{a}	4.8^{c}	5.0^{a}	4.8^{c}	5.1^{b}	4.8^{c}
2-Methyl-propanal	6.6^{a}	6.4^{b}	5.9^{f}	6.2^{d}	6.3^{c}	6.1^{e}	6.6^{a}	6.2^{d}	6.2^{d}	6.2^{d}
3-Methyl-butanal	7.0^{a}	6.9^{b}	6.6^{e}	6.8^{c}	6.8^{c}	6.5^{f}	7.0^{a}	6.7^{d}	6.7^{d}	6.9^{b}
2-Methyl-butanal	6.4^{a}	6.3^{b}	6.0^{e}	6.3^{b}	6.2^{c}	5.9^{f}	6.4^{a}	6.1^{d}	6.1^{d}	6.3^{b}
Benzaldehyde	6.8^{c}	6.7^{d}	6.5^{f}	6.8^{c}	6.6^{e}	6.3^{h}	7.0^{a}	6.4^{g}	6.6^{e}	6.9^{b}
Benzeneacetaldehyde	6.2^{b}	6.1^{c}	5.8^{f}	6.1^{c}	6.0^{d}	5.8^{f}	6.3^{a}	6.0^{d}	5.9^{e}	6.2^{b}
Alcohols										
Methanol	5.2^{c}	5.3^{c}	5.5^{a}	5.3^{c}	5.3^{c}	5.0^{d}	5.3^{c}	5.4^{b}	5.5^{a}	5.2^{c}
Ethanol	7.5^{c}	7.5^{c}	7.3^{e}	7.4^{d}	7.6^{b}	7.5^{c}	7.5^{c}	7.4^{d}	7.7^{a}	7.3^{e}
1-Propanol	5.9^{e}	6.1^{c}	6.0^{d}	6.0^{d}	6.1^{c}	6.2^{b}	6.0^{d}	6.0^{d}	6.3^{a}	6.1^{c}
1-Butanol	7.2^{d}	7.2^{d}	7.5^{b}	7.4^{c}	7.4^{c}	7.6^{a}	7.4^{c}	7.0^{f}	7.4^{c}	7.1^{e}
1-Pentanol	7.0^{a}	6.9^{b}	6.7^{d}	6.8^{c}	7.0^{a}	6.6^{e}	6.9^{b}	6.7^{d}	6.7^{d}	6.9^{b}
1-Hexanol	6.9^{e}	6.8^{f}	7.3^{b}	7.1^{c}	7.1^{c}	7.4^{a}	7.3^{b}	6.5^{g}	7.0^{d}	6.7^{g}
1-Heptanol	5.7^{a}	5.7^{a}	5.5^{c}	5.6^{b}	5.6^{b}	5.4^{d}	5.5^{c}	5.7^{a}	5.7^{a}	5.7^{a}
1-Octen-3-ol	5.3^{a}	5.2^{b}	4.8^{f}	5.3^{a}	5.0^{d}	4.4^{g}	4.9^{e}	5.0^{d}	4.9^{e}	5.1^{c}
2-Propanol	7.7^{b}	7.7^{b}	7.6^{c}	7.7^{b}	7.8^{a}	7.6^{c}	7.7^{b}	7.6^{c}	7.8^{a}	7.5^{d}
2-Butanol	6.7^{f}	7.1^{b}	6.8^{e}	6.7^{f}	7.0^{c}	6.9^{d}	6.7^{f}	7.1^{b}	7.3^{a}	7.0^{c}
2-Pentanol	7.0^{b}	7.0^{b}	7.1^{a}	7.1^{a}	7.1^{a}	6.7^{c}	7.0^{b}	7.0^{b}	7.1^{a}	7.0^{b}
2-Heptanol	7.4^{b}	7.5^{a}	7.5^{a}	7.4^{b}	7.5^{a}	6.9^{d}	7.2^{c}	7.5^{a}	7.4^{b}	7.4^{b}
2-Methyl-1-propanol	6.0^{a}	6.0^{a}	5.9^{b}	6.0^{a}	6.0^{a}	5.5^{e}	5.7^{d}	6.0^{a}	5.8^{c}	5.9^{b}
3-Methyl-3-buten-1-ol	5.5^{c}	5.5^{c}	5.5^{c}	5.5^{c}	5.6^{b}	5.6^{b}	5.6^{b}	5.4^{d}	5.7^{a}	5.5^{c}
3-Methyl-1-butanol	7.1^{b}	7.1^{b}	7.1^{b}	7.2^{a}	7.1^{b}	6.5^{e}	6.7^{f}	7.2^{a}	6.9^{d}	7.0^{c}
2-Methyl-1-butanol	6.3^{a}	6.3^{a}	6.3^{a}	6.3^{a}	6.3^{a}	5.8^{e}	5.9^{d}	6.3^{a}	6.1^{c}	6.2^{b}
2-Methyl-2-propanol	6.0^{b}	6.0^{b}	6.1^{a}	6.0^{b}	6.0^{b}	5.8^{d}	5.9^{c}	5.9^{c}	5.9^{c}	5.9^{c}

Alcohols										
3-Methyl-2-butanol	5.2^{c}	5.3^{b}	5.2^{c}	5.3^{b}	5.3^{b}	5.1^{d}	5.2^{c}	5.3^{b}	5.4^{a}	5.3^{b}
3-Methyl-2-pentanol	4.8^{b}	4.1^{d}	4.9^{a}	4.9^{a}	4.8^{b}	4.5^{d}	4.7^{c}	4.8^{b}	4.9^{a}	4.8^{b}
4-Methyl-2-pentanol	5.1^{b}	5.5^{a}	4.9^{d}	5.0^{c}	5.1^{b}	4.6^{d}	4.9^{d}	4.9^{d}	5.0^{c}	4.9^{d}
Ketones										
2-Propanone	7.8^{b}	7.9^{a}	7.6^{d}	7.7^{c}	7.8^{b}	7.4^{d}	7.7^{c}	7.8^{b}	7.8^{b}	7.7^{c}
2-Butanone	7.0^{d}	7.3^{b}	7.2^{c}	7.0^{d}	7.3^{b}	7.2^{c}	7.1^{d}	7.3^{b}	7.4^{a}	7.2^{c}
2-Pentanone	8.9^{b}	9.0^{a}	8.9^{b}	9.0^{a}	8.9^{b}	8.6^{d}	8.8^{c}	9.0^{a}	8.9^{b}	9.0^{a}
3-Hexanone	5.5^{a}	5.4^{b}	5.0^{d}	5.3^{c}	5.2^{d}	4.5^{f}	5.0^{d}	5.4^{b}	5.3^{c}	5.3^{c}
2-Hexanone	7.2^{c}	7.4^{a}	7.1^{d}	7.3^{b}	7.3^{b}	6.5^{f}	7.0^{d}	7.3^{b}	7.2^{c}	7.3^{b}
4-Heptanone	6.1^{a}	6.0^{b}	5.6^{d}	5.8^{c}	5.8^{c}	4.9^{d}	5.6^{d}	6.0^{b}	6.1^{a}	6.0^{b}
3-Heptanone	4.4^{a}	4.3^{b}	4.2^{c}	4.4^{a}	4.4^{a}	4.0^{d}	4.3^{b}	4.2^{c}	4.3^{b}	4.3^{b}
2-Heptanone	8.7^{c}	8.9^{a}	8.6^{d}	8.8^{b}	8.8^{b}	8.0^{f}	8.5^{d}	8.8^{b}	8.6^{d}	8.7^{c}
3-Octanone	5.4^{b}	5.4^{b}	5.1^{d}	5.5^{a}	5.3^{c}	4.6^{d}	5.1^{d}	5.4^{b}	5.4^{b}	5.3^{c}
2-Octanone	7.1^{c}	7.3^{a}	6.9^{d}	7.1^{c}	7.2^{b}	6.2^{g}	6.7^{f}	7.2^{b}	7.0^{d}	7.0^{d}
4-Nonanone	5.0^{c}	5.1^{b}	4.8^{d}	4.8^{d}	5.2^{a}	4.7^{f}	4.9^{d}	4.8^{d}	5.1^{b}	4.8^{d}
2-Nonanone	8.1^{c}	8.3^{a}	8.0^{d}	8.2^{b}	8.2^{b}	7.3^{g}	7.8^{f}	8.3^{a}	7.9^{d}	8.1^{c}
3-Buten-2-one	5.6^{a}	5.6^{a}	5.4^{d}	5.4^{d}	5.5^{c}	5.3^{d}	5.5^{c}	5.4^{d}	5.5^{c}	5.5^{c}
3-Penten-2-one	5.3^{c}	5.5^{a}	5.3^{c}	5.4^{b}	5.5^{a}	5.0^{d}	5.3^{c}	5.4^{b}	5.3^{c}	5.4^{b}
5-Hexen-2-one	5.9^{c}	6.1^{a}	5.7^{d}	5.8^{d}	5.9^{c}	5.4^{g}	5.6^{f}	6.0^{b}	6.1^{a}	5.9^{c}
6-Hepten-2-one	5.8^{a}	5.8^{a}	5.5^{d}	5.7^{b}	5.7^{b}	4.5^{f}	5.3^{d}	5.8^{a}	5.5^{d}	5.6^{c}
5-Hepten-2-one	4.2^{f}	4.7^{b}	4.3^{d}	4.3^{d}	4.6^{c}	0.0^{h}	3.9^{g}	5.1^{a}	4.6^{c}	4.5^{d}
1-Octen-3-one	5.5^{b}	5.5^{b}	5.3^{d}	5.3^{d}	5.4^{c}	5.3^{d}	5.6^{a}	5.3^{d}	5.5^{b}	5.3^{d}
8-Nonen-2-one	6.7^{c}	6.9^{a}	6.5^{d}	6.7^{c}	6.7^{c}	5.7^{g}	6.3^{f}	6.8^{b}	6.5^{d}	6.6^{d}
3-Hydroxy-2-butanone	5.9^{b}	5.8^{c}	5.7^{d}	5.8^{c}	5.6^{d}	5.4^{g}	5.5^{f}	5.6^{d}	6.0^{a}	5.9^{b}
2,3-Butanedione	7.5^{b}	7.5^{b}	7.5^{b}	7.5^{b}	7.5^{b}	7.3^{d}	7.4^{c}	7.4^{c}	7.5^{b}	7.6^{a}
2,3-Pentanedione	5.7^{b}	5.7^{b}	5.6^{c}	5.7^{b}	5.7^{b}	5.5^{d}	5.8^{a}	5.5^{d}	5.7^{b}	5.7^{b}
2,3-Hexanedione	4.4^{d}	4.6^{b}	4.4^{d}	4.7^{a}	4.6^{b}	4.3^{d}	4.6^{b}	4.5^{c}	4.4^{d}	4.4^{d}
2,3-Octanedione	5.8^{a}	5.6^{c}	5.4^{d}	5.4^{d}	5.6^{c}	5.6^{c}	5.8^{a}	5.4^{d}	5.7^{b}	5.5^{d}
3-Methyl-2-butanone	5.4^{c}	5.6^{a}	5.2^{d}	5.4^{c}	5.5^{b}	4.8^{f}	5.3^{d}	5.5^{b}	5.4^{c}	5.4^{c}
4-Methyl-2-pentanone	4.8^{c}	5.0^{a}	4.8^{c}	4.8^{c}	4.9^{b}	4.4^{d}	4.7^{d}	4.9^{b}	4.9^{b}	4.8^{c}
2-Methyl-3-pentanone	4.4^{d}	4.5^{c}	4.4^{d}	4.3^{d}	4.4^{d}	4.1^{f}	4.4^{d}	4.6^{b}	4.7^{a}	4.5^{c}
3-Methyl-2-pentanone	5.7^{c}	5.9^{a}	5.5^{d}	5.7^{c}	5.8^{b}	4.8^{g}	5.4^{f}	5.8^{b}	5.6^{d}	5.7^{c}
3-Methyl-2-heptanone	6.2^{c}	6.5^{a}	6.0^{d}	5.6^{f}	6.4^{b}	5.0^{h}	5.3^{g}	6.4^{b}	6.1^{d}	5.6^{f}
6-Methyl-3-heptanone	4.3^{d}	4.4^{c}	3.2^{h}	6.2^{a}	4.7^{g}	0.0^{i}	5.9^{b}	4.2^{d}	4.1^{f}	6.2^{a}
6-Methyl-2-heptanone	5.1^{b}	5.2^{a}	4.8^{d}	4.0^{g}	5.2^{a}	4.6^{d}	3.5^{h}	5.1^{b}	5.0^{c}	4.2^{f}
5-Methyl-2-heptanone	5.2^{b}	5.4^{a}	5.1^{c}	5.0^{d}	5.4^{a}	4.1^{f}	4.9^{d}	5.4^{a}	5.1^{c}	5.0^{d}
Cyclopentanone	4.8^{c}	4.7^{d}	4.5^{f}	5.3^{a}	4.5^{f}	4.4^{g}	4.8^{c}	4.6^{d}	4.7^{d}	5.2^{b}
1-Phenyl-ethanone	5.6^{b}	5.7^{a}	5.3^{d}	4.5^{f}	5.6^{b}	5.2^{d}	4.5^{f}	5.5^{c}	5.7^{a}	4.5^{f}
Esters										
Methyl acetate	5.3^{c}	5.5^{a}	5.2^{d}	5.3^{c}	5.4^{b}	5.1^{d}	5.3^{c}	5.2^{d}	5.2^{d}	5.1^{d}
Methyl butanoate	6.4^{b}	6.5^{a}	6.4^{b}	6.4^{b}	6.5^{a}	6.1^{d}	6.3^{c}	6.4^{b}	6.3^{c}	6.3^{c}
Methyl 3-methyl-butanoate	4.4^{c}	4.5^{b}	4.2^{d}	4.2^{d}	4.2^{d}	3.6^{g}	3.8^{f}	4.3^{d}	4.6^{a}	4.3^{d}
Methyl 2-methyl-butanoate	4.3^{c}	4.4^{b}	4.1^{d}	4.3^{c}	4.4^{b}	4.1^{d}	4.2^{d}	4.3^{c}	4.7^{a}	4.4^{b}
Esters										
Methyl pentanoate	4.7^{c}	4.8^{b}	4.7^{c}	5.0^{c}	4.8^{b}	4.6^{d}	4.7^{c}	4.7^{c}	4.6^{d}	4.8^{b}

Methyl hexanoate	6.4[d]	6.7[c]	6.5[c]	6.6[b]	6.6[b]	6.2[f]	6.3[e]	6.5[c]	6.4[d]	6.5[c]
Methyl octanoate	5.5[d]	5.8[c]	5.6[c]	5.7[b]	5.7[b]	5.4[e]	5.5[d]	5.7[b]	5.6[c]	5.7[b]
Methyl benzoate	5.3[c]	5.3[c]	5.2[b]	5.3[c]	5.3[c]	5.3[c]	5.3[c]	5.3[c]	5.3[c]	5.3[c]
Ethyl acetate	6.3[b]	6.4[c]	6.3[b]	6.4[c]	6.4[c]	6.3[b]	6.4[c]	6.2[c]	6.3[b]	6.3[b]
Ethyl propanoate	4.3[d]	4.4[c]	4.4[c]	6.4[c]	4.5[b]	4.3[d]	4.4[c]	4.3[d]	4.4[c]	4.3[d]
Ethyl 2-methyl-propionate	4.4[c]	4.5[b]	4.3[c]	6.4[c]	4.5[b]	4.1[e]	4.4[c]	4.3[c]	4.4[c]	4.4[c]
Ethyl butanoate	7.2[b]	7.2[b]	7.2[b]	6.4[c]	7.3[c]	7.3[c]	7.3[c]	7.1[c]	7.3[c]	7.2[b]
Ethyl 2-butenoate	4.2[b]	4.1[c]	3.7[f]	6.4[c]	4.0[c]	3.3[g]	3.2[h]	3.8[e]	3.8[e]	3.7[f]
Ethyl 2-methyl-butanoate	4.3[c]	4.4[b]	4.1[e]	6.4[c]	4.3[c]	3.2[g]	3.9[f]	4.2[c]	4.2[c]	4.2[c]
Ethyl 3-methyl-butanoate	4.3[c]	4.4[b]	4.2[c]	6.4[c]	4.3[c]	3.5[f]	4.1[e]	4.3[c]	4.3[c]	4.3[c]
Ethyl pentanoate	5.1[c]	5.1[c]	5.1[c]	6.4[c]	5.2[b]	5.2[b]	5.2[b]	5.0[c]	5.2[b]	5.1[c]
Ethyl hexanoate	7.0[c]	7.1[b]	7.1[b]	6.4[c]	7.1[b]	7.2[c]	7.1[b]	7.0[c]	7.2[c]	7.1[b]
Ethyl heptanoate	4.6[e]	4.8[c]	4.8[c]	6.4[c]	4.9[b]	4.9[b]	4.8[c]	4.7[c]	4.9[b]	4.8[c]
Ethyl octanoate	5.6[c]	5.8[b]	5.8[b]	5.8[b]	5.9[c]	5.9[c]	5.8[b]	5.7[c]	5.8[b]	5.9[c]
Propyl acetate	5.4[c]	5.6[b]	5.6[b]	5.7[c]	5.6[b]	5.2[f]	5.3[e]	5.5[c]	5.4[c]	5.4[c]
Propyl butanoate	5.0[e]	5.3[b]	5.1[c]	5.2[c]	5.2[c]	5.0[e]	5.1[c]	5.2[c]	5.5[c]	5.2[c]
Propyl hexanoate	4.4[c]	4.6[c]	4.5[b]	4.5[b]	4.5[b]	4.5[b]	4.4[c]	4.5[b]	4.6[c]	4.5[b]
Butyl acetate	5.7[c]	5.7[c]	6.2[c]	5.9[b]	5.9[b]	6.2[c]	6.1[b]	5.3[e]	5.7[c]	5.5[c]
Butyl butanoate	5.0[f]	5.1[e]	5.5[b]	5.3[c]	5.3[c]	5.5[b]	5.4[c]	5.1[e]	5.6[c]	4.9[g]
Pentyl acetate	4.9[b]	5.0[c]	4.8[c]	4.9[b]	4.9[b]	4.1[e]	4.5[c]	5.0[c]	4.8[c]	4.8[c]
2-Pentyl butanoate	4.5[c]	4.8[b]	4.8[b]	3.9[f]	4.9[c]	4.2[e]	3.4[h]	4.7[c]	4.9[c]	3.6[g]
Hexyl acetate	4.1[c]	4.3[c]	4.2[b]	4.1[c]	4.3[c]	4.1[c]	4.2[b]	3.7[e]	4.2[b]	3.9[c]
3-Methyl-butyl acetate	5.3[b]	5.4[c]	5.4[c]	4.9[e]	5.3[b]	4.9[e]	4.9[e]	5.2[c]	5.1[c]	4.9[e]
2-Methyl-butyl acetate	4.9[c]	4.6[c]	4.4[e]	4.4[e]	4.7[b]	4.1[h]	4.0[g]	4.5[c]	4.6[c]	4.3[f]
1-Methyl-propyl butanoate	4.5[e]	4.9[c]	4.4[f]	5.4[c]	4.9[c]	4.3[g]	5.1[b]	4.7[c]	4.7[c]	5.1[b]
2-Methyl-propyl butanoate	5.2[b]	5.4[c]	5.1[c]	4.6[c]	5.4[c]	4.6[c]	4.5[e]	5.2[b]	5.1[c]	4.4[f]
Isopropyl butanoate	5.0[c]	5.0[c]	4.9[b]	4.5[c]	5.0[c]	4.6[c]	4.2[e]	4.9[b]	5.0[c]	4.5[c]
2-Methyl-propyl acetate	4.5[c]	4.5[c]	4.2[g]	5.3[c]	4.4[e]	3.2[h]	5.0[c]	4.3[f]	4.4[e]	5.2[b]
3-Methyl-butyl butanoate	5.3[c]	5.5[c]	5.2[c]	5.4[b]	5.5[c]	4.8[e]	5.1[c]	5.4[b]	5.2[c]	5.2[c]
2-Methyl-butyl butanoate	4.8[c]	5.2[c]	4.6[e]	4.8[c]	5.0[b]	4.2[c]	4.5[f]	4.7[c]	4.8[c]	4.7[c]
Sulfur compounds										
Methanethiol	5.3[b]	4.9[c]	4.2[g]	4.7[c]	4.6[e]	4.5[f]	5.4[c]	4.5[f]	4.9[c]	4.9[c]
Dimethyl-sulfide	4.2[f]	4.9[b]	4.0[g]	4.7[c]	5.0[c]	4.4[e]	4.5[c]	4.0[g]	4.5[c]	4.0[g]
Dimethyl-disulfide	6.5[b]	6.3[c]	5.4[h]	6.0[e]	5.6[f]	5.3[i]	7.0[c]	5.5[g]	6.1[c]	6.0[e]
Dimethyl-trisulfide	5.0[b]	4.4[c]	3.9[i]	4.1[g]	4.2[f]	4.0[h]	4.9[c]	4.0[h]	5.2[c]	4.3[e]
2,4-Dithiapentane	4.1[c]	4.1[c]	4.1[c]	3.6[e]	4.1[c]	4.1[c]	4.5[c]	3.9[c]	4.2[b]	4.1[c]
Methional	5.1[c]	4.6[c]	3.9[g]	4.1[f]	4.2[e]	4.3[c]	4.1[f]	4.3[c]	4.8[b]	3.9[g]
S-methyl thioacetate	4.7[c]	4.6[c]	4.7[c]	4.5[e]	4.6[c]	4.8[b]	5.2[c]	4.3[f]	4.7[c]	4.6[c]
Thiophene	5.4[c]	5.6[c]	5.4[c]	4.7[e]	5.5[b]	4.9[c]	4.9[c]	5.4[c]	5.5[b]	4.6[f]
2-Methyl-tetrahydro-thiophen-3-one	4.5[c]	4.3[e]	4.1[g]	5.5[c]	4.2[f]	3.7[h]	5.4[b]	4.3[e]	4.6[c]	5.4[b]
Methyl isothiocyanate	6.0[c]	5.5[b]	4.6[f]	4.3[g]	5.1[c]	4.7[e]	4.1[h]	4.7[e]	5.3[c]	4.3[g]
Furans										
2-Methyl-furan	5.2[a]	5.1[b]	4.9[c]	5.1[b]	5.1[b]	4.7[e]	5.0[c]	5.0[c]	5.0[c]	5.1[b]
2-Ethyl-furan	5.7[b]	5.7[c]	5.6[c]	5.6[c]	5.8[a]	5.6[c]	5.8[a]	5.6[c]	5.8[a]	5.7[b]
2-Propyl-furan	4.7[b]	4.8[a]	4.6[c]	4.6[c]	4.8[a]	4.7[b]	4.7[b]	4.8[a]	4.7[b]	4.7[b]
3-Methyl-furan	4.7[a]	4.6[b]	4.4[c]	4.6[b]	4.6[b]	4.4[c]	4.7[a]	4.6[b]	4.5[c]	4.7[a]
2,4-Dimethyl-furan	4.7[c]	4.6[c]	4.5[e]	4.6[c]	4.6[c]	4.4[f]	4.3[g]	5.3[a]	4.8[b]	4.7[c]

2,5-Dimethyl-furan	4.6^{c}	4.7^{b}	4.7^{b}	4.7^{b}	4.7^{b}	4.5^{c}	3.9^{e}	4.8^{a}	4.7^{b}	4.7^{b}
Furfural	5.0^{a}	4.8^{c}	4.6^{c}	4.9^{b}	4.9^{b}	4.6^{c}	4.9^{b}	4.8^{c}	4.6^{c}	4.8^{c}
	**Pecorino Siciliano**									
Aldehydes										
Acetaldehyde	5.9^{d}	6.0^{c}	6.4^{a}	6.2^{b}	6.0^{c}	6.0^{c}	6.2^{b}	5.9^{d}	6.0^{c}	6.0^{c}
Butanal	5.9^{g}	6.1^{e}	6.4^{b}	6.2^{d}	6.0	6.6^{a}	6.3^{c}	5.7^{i}	6.0^{f}	5.8^{h}
Pentanal	6.3^{f}	6.8^{b}	6.6^{d}	6.2^{g}	6.3^{f}	6.4^{e}	7.1^{a}	6.3^{f}	6.4^{e}	6.7^{c}
Hexanal	6.5^{c}	6.6^{b}	6.6^{b}	6.2^{e}	6.5^{c}	6.7^{a}	6.7^{a}	6.5^{c}	6.6^{b}	6.3^{d}
Heptanal	5.4^{f}	5.7^{c}	5.7^{c}	6.2^{a}	5.3^{g}	5.3^{g}	6.0^{b}	5.4^{f}	5.5^{e}	5.6^{d}
Octanal	5.6^{e}	5.8^{d}	5.9^{c}	6.2^{a}	5.5^{f}	5.8^{d}	6.0^{b}	5.4^{g}	5.6^{e}	5.6^{e}
Nonanal	6.2^{b}	6.3^{a}	6.2^{b}	6.2^{b}	5.9^{e}	5.9^{e}	6.3^{a}	5.9^{e}	6.0^{d}	6.1^{c}
Decanal	4.5^{e}	4.7^{c}	4.5^{e}	6.2^{a}	4.3^{f}	4.5^{e}	4.8^{b}	4.2^{g}	4.6^{d}	4.5^{e}
Propenal	5.4^{f}	5.7^{d}	6.0^{b}	6.2^{a}	5.5^{e}	6.0^{b}	5.8^{c}	5.1^{h}	5.7^{d}	5.2^{g}
2-Pentenal	5.0^{d}	5.3^{c}	5.4^{h}	6.2^{a}	4.8^{f}	4.9^{e}	5.8^{b}	4.7^{g}	5.0^{d}	5.0^{d}
2-Hexenal	4.8^{d}	5.1^{c}	5.1^{c}	6.2^{a}	4.7^{e}	4.7^{e}	5.5^{b}	4.6^{f}	4.8^{d}	4.8^{d}
2,4-Hexadienal	4.4^{f}	4.7^{c}	4.6^{d}	6.2^{a}	4.6^{d}	4.0^{g}	5.1^{b}	4.0^{g}	4.5^{e}	4.5^{e}
2-Heptenal	5.0^{e}	5.4^{c}	5.4^{c}	6.2^{a}	4.8^{f}	4.8^{f}	5.9^{b}	4.8^{f}	5.1^{d}	5.1^{d}
2-Octenal	4.8^{e}	5.1^{c}	5.1^{c}	6.2^{a}	4.5^{h}	4.7^{f}	5.6^{b}	4.6^{g}	4.9^{d}	4.7^{f}
2-Methyl-propanal	6.0^{e}	6.4^{c}	6.5^{b}	6.2^{d}	6.0^{e}	5.8^{g}	6.6^{a}	5.9^{f}	6.0^{e}	6.2^{d}
3-Methyl-butanal	6.2^{e}	6.6^{b}	6.5^{c}	6.2^{e}	6.2^{e}	6.1^{f}	6.8^{a}	6.3^{d}	6.6^{b}	6.5^{c}
2-Methyl-butanal	6.2^{e}	6.6^{b}	6.5^{c}	6.2^{e}	6.2^{e}	6.2^{e}	6.7^{a}	6.2^{e}	6.4^{d}	6.4^{d}
Benzaldehyde	6.4^{e}	6.8^{b}	6.7^{c}	6.2^{f}	6.2^{f}	6.2^{f}	6.9^{a}	6.2^{f}	6.6^{d}	6.6^{d}
Benzeneacetaldehyde	5.6^{e}	6.1^{c}	6.1^{c}	6.2^{b}	5.3^{f}	5.6^{e}	6.4^{a}	5.2^{h}	5.8^{d}	5.4^{g}
Alcohols										
Methanol	4.7^{g}	4.8^{f}	4.9^{e}	4.9^{e}	5.9^{a}	5.4^{b}	5.0^{d}	5.1^{c}	5.0^{d}	5.1^{c}
Ethanol	6.9^{c}	7.0^{b}	7.1^{a}	7.0^{b}	7.0^{b}	7.1^{a}	6.9^{c}	7.0^{b}	7.1^{a}	6.9^{c}
1-Propanol	6.6^{e}	6.6^{e}	7.0^{b}	6.6^{e}	6.7^{d}	7.1^{a}	6.6^{e}	6.3^{f}	6.8^{c}	6.2^{g}
1-Butanol	7.1^{f}	7.1^{f}	7.5^{b}	7.3^{d}	7.3^{d}	7.8^{a}	7.2^{e}	6.8^{g}	7.4^{c}	6.7^{h}
1-Pentanol	6.4^{e}	6.6^{c}	6.4^{e}	6.5^{d}	6.3^{f}	6.3^{f}	6.8^{b}	6.8^{b}	7.1^{a}	6.8^{b}
1-Hexanol	6.8^{e}	6.7^{f}	7.0^{c}	6.7^{f}	6.9^{d}	7.6^{a}	6.9^{d}	6.5^{g}	7.1^{b}	6.4^{h}
1-Heptanol	5.8^{e}	5.9^{d}	5.7^{f}	5.8^{e}	5.6^{g}	5.7^{f}	5.9^{d}	6.2^{b}	6.5^{a}	6.1^{c}
1-Octen-3-ol	4.3^{e}	4.5^{d}	4.3^{e}	4.3^{e}	4.3^{e}	4.1^{f}	4.7^{b}	4.5^{d}	4.9^{a}	4.6^{c}
2-Propanol	7.3^{d}	7.2^{e}	7.4^{c}	7.2^{e}	7.3^{d}	7.6^{b}	7.2^{e}	7.4^{c}	7.7^{a}	7.0^{f}
2-Butanol	7.9^{f}	7.9^{f}	8.3^{b}	7.8^{g}	8.1^{d}	8.5^{a}	8.0^{e}	7.6^{h}	8.2^{c}	7.5^{i}
2-Pentanol	6.7^{d}	6.6^{e}	6.5^{f}	6.3^{g}	6.8^{c}	6.7^{d}	6.7^{d}	7.0^{b}	7.1^{a}	6.5^{f}
Alcohols										
2-Heptanol	7.2^{d}	7.3^{c}	7.1^{e}	6.9^{f}	7.3^{c}	7.1^{e}	7.3^{c}	7.7^{a}	7.6^{b}	7.3^{c}
2-Methyl-1-propanol	5.6^{b}	5.7^{a}	5.6^{b}	5.5^{c}	5.5^{c}	5.5^{c}	5.6^{b}	5.4^{d}	5.5^{c}	5.4^{d}
3-Methyl-3-buten-1-ol	5.4^{b}	5.3^{c}	5.4^{b}	5.3^{c}	5.4^{b}	5.5^{a}	5.4^{b}	5.3^{c}	5.5^{a}	5.2^{d}
3-Methyl-1-butanol	7.0^{b}	7.1^{a}	7.0^{b}	7.0^{b}	6.9^{c}	6.8^{d}	7.0^{b}	6.7^{e}	6.8^{d}	6.7^{e}
2-Methyl-1-butanol	6.2^{b}	6.3^{a}	6.3^{a}	6.2^{b}	6.0^{d}	6.2^{b}	6.1^{c}	6.0^{d}	6.1^{c}	5.9^{e}
2-Methyl-2-propanol	5.3^{b}	5.3^{b}	5.3^{b}	4.8^{f}	5.1^{d}	5.2^{c}	5.0^{e}	5.5^{a}	5.3^{b}	5.2^{c}
3-Methyl-2-butanol	5.1^{c}	5.1^{c}	5.1^{c}	5.0^{d}	5.0^{d}	5.3^{a}	5.1^{c}	5.2^{b}	5.2^{b}	4.9^{e}
3-Methyl-2-pentanol	4.7^{d}	4.7^{d}	4.4^{f}	4.6^{e}	4.6^{e}	4.1^{g}	4.7^{d}	5.3^{a}	5.1^{b}	4.9^{c}
4-Methyl-2-pentanol	4.8^{d}	4.9^{c}	4.9^{c}	4.8^{d}	4.8^{d}	4.9^{c}	5.0^{b}	5.4^{a}	4.9^{c}	5.4^{a}
2-Propanone	7.6^{d}	7.6^{d}	7.4^{e}	7.4^{e}	7.4^{e}	6.8^{f}	7.7^{c}	7.7^{c}	7.9^{a}	7.8^{b}
2-Butanone	7.8^{b}	7.8^{b}	7.9^{a}	7.7^{c}	7.8^{b}	7.7^{c}	7.8^{b}	7.6^{c}	7.9^{a}	7.6^{c}
2-Pentanone	8.8^{d}	8.9^{c}	8.5^{f}	8.6^{e}	8.8^{d}	7.6^{g}	8.9^{c}	8.9^{c}	9.1^{a}	9.0^{b}

Ketones										
3-Hexanone	5.2^{c}	5.4^{a}	5.3^{b}	5.3^{b}	5.1^{d}	4.6^{e}	5.2^{c}	5.2^{c}	5.3^{b}	5.3^{b}
2-Hexanone	4.8^{f}	5.1^{d}	5.1^{d}	4.8^{f}	4.9^{e}	5.2^{c}	7.0^{b}	4.9^{e}	5.1^{c}	7.3^{a}
4-Heptanone	4.2^{e}	4.4^{c}	4.4^{c}	4.4^{c}	4.2^{e}	4.2^{e}	5.0^{a}	4.3^{d}	4.6^{b}	5.0^{a}
3-Heptanone	8.5^{d}	8.6^{c}	8.1^{g}	8.3^{f}	8.4^{e}	7.3^{h}	4.4^{j}	8.7^{b}	8.9^{a}	4.5^{i}
2-Heptanone	4.9^{g}	5.2^{d}	4.9^{g}	4.9^{g}	5.0^{f}	4.7^{h}	8.5^{b}	5.1^{e}	5.3^{c}	8.7^{a}
3-Octanone	6.8^{c}	6.8^{c}	6.3^{f}	6.5^{e}	6.6^{d}	5.6^{g}	5.1^{h}	7.1^{b}	7.4^{a}	5.1^{h}
2-Octanone	4.7^{e}	5.1^{c}	4.8^{d}	4.5^{f}	4.4^{g}	4.7^{e}	6.8^{b}	4.4^{g}	4.7^{e}	7.1^{a}
4-Nonanone	8.1^{c}	8.1^{c}	7.6^{f}	7.7^{e}	7.9^{d}	7.1^{g}	5.0^{h}	8.4^{b}	8.7^{a}	4.4^{i}
2-Nonanone	5.4^{e}	5.6^{c}	5.4^{e}	5.5^{d}	5.3^{f}	5.3^{f}	8.1^{b}	5.3^{f}	5.5^{d}	8.4^{a}
3-Buten-2-one	5.2^{d}	5.2^{d}	4.9^{e}	4.9^{e}	4.9^{e}	4.7^{f}	5.6^{a}	5.2^{d}	5.3^{c}	5.5^{b}
3-Penten-2-one	5.6^{c}	5.6^{c}	5.3^{e}	5.3^{e}	5.5^{d}	5.1^{g}	5.2^{f}	6.1^{b}	6.7^{a}	5.3^{e}
5-Hexen-2-one	6.2^{b}	5.9^{c}	5.4^{f}	5.5^{e}	5.5^{e}	4.5^{g}	5.6^{d}	6.4^{a}	6.4^{a}	6.2^{b}
6-Hepten-2-one	4.8^{e}	4.4^{f}	3.2^{i}	3.6^{h}	4.0^{g}	0.0^{j}	5.8^{b}	4.9^{d}	5.0^{c}	5.9^{a}
5-Hepten-2-one	4.9^{c}	5.1^{a}	5.0^{b}	5.1^{a}	4.9^{c}	4.8^{d}	4.2^{f}	4.7^{e}	5.1^{a}	5.0^{b}
1-Octen-3-one	6.6^{d}	6.7^{c}	6.1^{g}	6.2^{f}	6.4^{e}	5.3^{i}	5.3^{i}	6.9^{b}	7.2^{a}	5.0^{h}
8-Nonen-2-one	5.4^{f}	5.6^{d}	5.2^{g}	5.5^{e}	5.2^{g}	4.4^{h}	6.6^{b}	5.2^{g}	5.7^{c}	6.9^{a}
3-Hydroxy-2-butanone	7.0^{c}	7.1^{b}	7.0^{c}	7.1^{b}	6.8^{d}	6.7^{e}	5.6^{f}	7.1^{b}	7.2^{a}	5.2^{g}
2,3-Butanedione	6.1^{c}	6.1^{c}	6.0^{d}	6.1^{c}	6.0^{d}	5.6^{e}	7.0^{b}	6.0^{d}	6.0^{d}	7.3^{a}
2,3-Pentanedione	4.2^{f}	4.1^{g}	4.5^{c}	4.4^{d}	4.2^{f}	4.3^{e}	6.2^{a}	4.2^{f}	4.4^{d}	6.1^{b}
2,3-Hexanedione	5.2^{c}	5.5^{a}	5.3^{b}	5.3^{b}	4.9^{e}	4.9^{e}	4.4^{f}	5.0^{d}	5.2^{c}	4.4^{f}
2,3-Octanedione	5.4^{c}	5.4^{c}	5.1^{e}	5.2^{d}	5.2^{d}	4.6^{f}	5.6^{b}	5.4^{c}	5.7^{a}	5.4^{c}
3-Methyl-2-butanone	4.8^{e}	4.8^{e}	4.7^{f}	4.6^{g}	4.7^{f}	4.4^{h}	5.4^{c}	4.9^{d}	5.0^{b}	5.6^{a}
4-Methyl-2-pentanone	4.2^{f}	4.4^{c}	4.4^{c}	4.4^{c}	4.3^{e}	4.3	4.7^{b}	4.4^{d}	4.5^{c}	4.9^{a}
2-Methyl-3-pentanone	5.7^{b}	5.9^{a}	5.4^{d}	5.6^{c}	5.6^{c}	4.4^{f}	4.3^{g}	5.9^{a}	5.9^{a}	4.6^{e}
3-Methyl-2-pentanone	4.9^{f}	5.1^{e}	4.7^{g}	4.5^{h}	4.9^{f}	3.7^{i}	5.8^{b}	5.3^{d}	5.6^{c}	5.9^{a}
3-Methyl-2-heptanone	5.7^{d}	5.9^{c}	5.4^{f}	5.5^{e}	5.7^{d}	4.4^{h}	4.8^{g}	6.4^{b}	6.6^{a}	4.8^{g}
6-Methyl-3-heptanone	4.1^{g}	4.5^{c}	4.3^{e}	4.3^{e}	4.2^{f}	4.2^{f}	5.9^{b}	4.2^{f}	4.4^{d}	6.2^{a}
6-Methyl-2-heptanone	4.8^{d}	5.1^{b}	4.9^{c}	4.9^{c}	4.7^{e}	4.7^{e}	4.2^{f}	4.9^{c}	5.3^{a}	3.8^{g}
5-Methyl-2-heptanone	4.8^{e}	4.9^{d}	4.6^{f}	4.6^{f}	4.6^{f}	3.9^{g}	5.1^{c}	5.2^{b}	5.6^{a}	4.9^{d}
Cyclopentanone	4.8^{d}	5.2^{a}	5.0^{b}	4.8^{d}	4.7^{e}	4.6^{f}	4.8^{d}	4.7^{e}	4.9^{c}	5.2^{a}
Ketones										
1-Phenyl-ethanone	5.8^{c}	6.0^{b}	5.7^{d}	5.7^{d}	5.7^{d}	5.6^{e}	5.1^{f}	5.8^{c}	6.1^{a}	5.1^{f}
Esters										
Methyl acetate	5.0^{c}	5.0^{c}	5.0^{c}	5.0^{c}	4.8^{d}	4.7^{e}	5.3^{a}	5.0^{c}	5.1^{b}	4.8^{d}
Methyl butanoate	6.1^{c}	6.2^{b}	6.1^{c}	6.0^{d}	6.2^{b}	6.3^{a}	6.2^{b}	6.1^{c}	6.3^{a}	5.9^{e}
Methyl 3-methyl-butanoate	4.7^{b}	4.4^{c}	4.2^{d}	3.9^{f}	3.7^{g}	3.1^{h}	4.4^{c}	4.7^{b}	4.8^{a}	4.1^{e}
Methyl 2-methyl-butanoate	4.2^{c}	4.3^{b}	4.0^{d}	3.7^{f}	3.0^{h}	3.3^{g}	4.4^{a}	3.3^{g}	3.9^{e}	3.7^{f}
Methyl pentanoate	4.6^{d}	4.5^{e}	4.6^{d}	4.6^{d}	4.7^{c}	4.9^{b}	4.6^{d}	4.6^{d}	5.1^{a}	4.6^{d}
Methyl hexanoate	6.0^{e}	6.2^{c}	6.1^{d}	5.9^{f}	6.2^{c}	6.5^{a}	6.2^{c}	6.0^{e}	6.3^{b}	5.8^{g}
Methyl octanoate	5.0^{d}	5.2^{b}	5.0^{d}	4.9^{e}	5.1^{c}	5.4^{a}	5.2^{b}	5.1^{c}	5.4^{a}	5.0^{d}
Methyl benzoate	5.1^{a}	5.1^{a}	5.0^{b}	5.0^{b}	5.0^{b}	5.0^{b}	5.1^{a}	4.9^{c}	5.1^{a}	5.0^{b}
Ethyl acetate	6.9^{b}	7.1^{a}	7.1^{a}	6.9^{b}	6.8^{c}	6.9^{b}	6.9^{b}	6.7^{d}	6.8^{c}	6.7^{d}
Ethyl propanoate	5.7^{b}	5.5^{c}	5.4^{d}	5.4^{d}	5.5^{c}	5.5^{c}	5.7^{b}	5.4^{d}	5.8^{a}	5.3^{e}
Ethyl 2-methyl-propionate	5.1^{b}	5.2^{a}	4.9^{e}	5.2^{a}	5.0^{d}	4.8^{f}	5.2^{a}	5.1^{b}	5.1^{b}	4.9^{e}
Ethyl butanoate	7.5^{c}	7.6^{b}	7.5^{c}	7.5^{c}	7.6^{b}	7.7^{a}	7.6^{b}	7.6^{b}	7.6^{b}	7.2^{d}
Ethyl 2-butenoate	5.2^{b}	5.1^{c}	4.6^{f}	4.8^{e}	5.2^{b}	3.3^{g}	5.2^{b}	5.7^{a}	5.7^{a}	5.0^{d}
Ethyl 2-methyl-butanoate	4.8^{b}	4.9^{a}	4.7^{c}	4.9^{a}	4.8^{b}	4.9^{a}	4.9^{a}	4.7^{c}	4.7^{c}	4.5^{d}

Ethyl 3-methyl-butanoate	5.2^{c}	5.2^{c}	4.9^{e}	5.4^{b}	4.9^{e}	4.9^{e}	5.5^{a}	5.4^{b}	5.5^{a}	5.0^{d}
Ethyl pentanoate	5.3^{g}	5.5^{e}	5.4^{f}	7.4^{b}	5.4^{f}	5.8^{d}	7.5^{a}	5.4^{f}	5.5^{e}	7.0^{c}
Ethyl hexanoate	7.4^{d}	7.5^{c}	7.5^{c}	4.8^{f}	7.5^{c}	7.8^{a}	5.0^{e}	7.4^{d}	7.6^{b}	4.6^{g}
Ethyl heptanoate	4.8^{h}	5.0^{f}	4.9^{g}	6.0^{b}	5.0^{f}	5.3^{d}	6.1^{a}	4.9^{g}	5.1^{e}	5.8^{c}
Ethyl octanoate	5.9^{e}	6.1^{c}	5.9^{e}	5.5^{f}	6.0^{d}	6.3^{a}	5.5^{f}	6.1^{c}	6.2^{b}	6.1^{c}
Propyl acetate	5.4^{g}	5.6^{f}	5.7^{e}	6.4^{a}	5.4	5.9^{c}	6.4^{a}	5.7^{e}	6.3^{b}	5.8^{d}
Propyl butanoate	6.2^{e}	6.3^{d}	6.6^{b}	5.6^{h}	6.4^{c}	7.0^{a}	5.7^{g}	6.0^{f}	6.6^{b}	5.0^{i}
Propyl hexanoate	5.6^{d}	5.6^{d}	5.9^{b}	5.8^{c}	5.8^{c}	6.5^{a}	5.8^{c}	5.3^{e}	5.8^{c}	5.8^{c}
Butyl acetate	5.8^{e}	5.7^{f}	6.0^{c}	6.0^{c}	5.8^{e}	6.6^{a}	5.9^{d}	5.6^{g}	6.3^{b}	5.4^{h}
Butyl butanoate	5.8^{e}	5.9^{d}	6.2^{c}	4.8^{h}	5.9^{d}	6.7^{a}	5.0^{g}	5.6^{f}	6.2^{c}	6.3^{b}
Pentyl acetate	5.3^{c}	5.1^{d}	4.8^{f}	5.0^{e}	4.5^{g}	4.2^{h}	5.3^{c}	6.1^{b}	6.7^{a}	5.0^{e}
2-Pentyl butanoate	4.9^{g}	5.4^{c}	5.0^{f}	4.5^{i}	5.1^{e}	5.2^{d}	4.3_{j}	5.7^{a}	5.6^{b}	4.6^{h}
Hexyl acetate	4.3^{g}	4.3^{g}	5.2^{c}	5.3^{b}	4.0^{i}	4.6^{f}	5.4^{a}	4.4^{h}	5.0^{d}	4.9^{e}
3-Methyl-butyl acetate	5.5^{d}	5.8^{b}	5.9^{a}	4.9^{f}	5.4^{e}	5.7^{c}	4.6^{g}	5.4^{e}	5.5^{d}	4.5^{h}
2-Methyl-butyl acetate	4.6^{d}	5.2^{c}	5.3^{b}	5.7^{a}	4.4^{f}	4.4^{f}	5.7^{a}	4.5^{e}	4.4^{f}	5.3^{b}
1-Methyl-propyl butanoate	6.1^{e}	6.0^{f}	6.6^{c}	4.9^{h}	6.4^{d}	7.1^{a}	4.9^{h}	5.8^{g}	6.7^{b}	4.5^{i}
2-Methyl-propyl butanoate	5.3^{f}	5.6^{c}	5.5^{d}	6.1^{b}	5.4^{e}	5.5^{d}	6.3^{a}	5.3^{f}	5.6^{c}	5.3^{f}
Isopropyl butanoate	5.2^{g}	5.4^{e}	5.4^{e}	5.5^{d}	5.3^{f}	5.7^{b}	5.6^{c}	5.8^{a}	5.8^{a}	5.1^{h}
2-Methyl-propyl acetate	4.5^{f}	5.0^{e}	5.2^{d}	5.8^{b}	4.1^{i}	3.5_{j}	5.9^{a}	4.3^{g}	4.2^{h}	5.3^{c}
3-Methyl-butyl butanoate	5.7^{c}	5.9^{b}	5.9^{b}	5.3^{e}	5.7^{c}	6.0^{a}	5.3^{e}	5.6^{d}	5.9^{b}	4.9^{b}
2-Methyl-butyl butanoate	5.2^{e}	5.6^{a}	5.4^{c}	5.3^{d}	5.1^{f}	5.5^{b}	5.3^{d}	5.3^{d}	5.4^{c}	4.9^{g}
Sulfur compounds										
Methanethiol	4.8^{f}	5.4^{c}	5.7^{b}	5.2^{d}	4.6^{h}	4.8^{f}	5.9^{a}	4.3^{i}	5.1^{e}	4.7^{g}
Dimethyl-sulfide	4.5^{b}	4.4^{c}	4.6^{a}	4.1^{f}	4.4^{c}	4.5^{b}	4.6^{a}	4.3^{d}	4.4^{c}	4.2^{e}
Dimethyl-disulfide	6.8^{f}	7.0^{d}	7.4^{a}	7.3^{b}	6.6^{h}	$6.^{H}6$	7.3^{b}	6.7^{g}	6.9^{e}	7.1^{c}
Dimethyl-trisulfide	5.0^{f}	5.7^{b}	5.7^{b}	6.1^{a}	4.9^{g}	4.9^{g}	5.6^{c}	4.9^{g}	5.1^{e}	5.2^{d}
2,4-Dithiapentane	5.0^{c}	5.0^{c}	5.0^{c}	5.1^{b}	5.0^{c}	5.3^{a}	5.1^{b}	4.9^{d}	5.1^{b}	4.9^{d}
Methional	5.0^{b}	4.4^{f}	5.0^{b}	4.7^{d}	4.9^{c}	5.2^{a}	4.6^{e}	4.7^{d}	4.6^{e}	4.4^{f}
S-methyl thioacetate	4.8^{e}	5.5^{c}	5.6^{b}	5.5^{c}	5.0^{d}	5.0^{d}	6.1^{a}	4.2^{g}	5.0^{d}	4.6^{f}
Thiophene	5.2^{e}	5.4^{d}	5.7^{b}	6.6^{a}	5.0^{g}	5.5^{c}	5.2^{e}	4.8^{h}	5.1^{f}	5.0^{g}
2-Methyl-tetrahydro-thiophen-3-one	4.6^{f}	5.0^{b}	4.9^{c}	4.8^{d}	4.3^{h}	4.5^{g}	5.2^{a}	4.6^{f}	4.8^{d}	4.7^{e}
Methyl isothiocyanate	5.0^{b}	5.0^{b}	5.1^{a}	4.9^{c}	4.9^{c}	5.1^{a}	5.0^{b}	4.7^{e}	5.0^{b}	4.8^{d}
Furans										
2-Methyl-furan	5.0^{c}	5.2^{a}	5.0^{c}	5.1^{b}	5.0^{c}	5.2^{a}	5.2^{a}	5.0^{c}	5.2^{a}	5.0^{c}
2-Ethyl-furan	5.5^{e}	5.8^{b}	5.8^{b}	5.6^{d}	5.4^{f}	5.5^{e}	6.1^{a}	5.5^{e}	5.7^{c}	5.6^{d}
2-Propyl-furan	4.5^{d}	4.8^{c}	4.9^{b}	4.6^{c}	4.4^{e}	4.4^{e}	5.1^{a}	4.4^{e}	4.6^{c}	4.5^{d}
3-Methyl-furan	4.6^{d}	4.9^{a}	4.7^{c}	4.7^{c}	4.5^{e}	4.7^{c}	4.9^{a}	4.5^{e}	4.8^{b}	4.6^{d}
2,4-Dimethyl-furan	4.8^{b}	4.9^{a}	4.7^{c}	4.9^{a}	4.7^{c}	4.7^{c}	4.4^{e}	4.7^{c}	4.8^{b}	4.5^{d}
2,5-Dimethyl-furan	4.8^{b}	4.7^{c}	4.6^{d}	4.4^{f}	4.4^{f}	4.7^{c}	4.6^{d}	4.5^{e}	4.9^{a}	4.7^{c}
Furfural	4.8^{c}	5.0^{b}	5.0^{b}	4.8^{c}	4.6^{e}	4.7^{d}	5.1^{a}	4.6^{e}	4.8^{c}	4.6^{e}
	**Pecorino Toscano**									
Acetaldehyde	5.9^{b}	5.9^{b}	5.9^{b}	5.9^{b}	6.0^{a}	6.0^{a}	6.0^{a}	5.9^{b}	5.8^{c}	6.0^{a}
Butanal	5.4^{c}	5.4^{c}	5.7^{b}	5.7^{b}	5.8^{a}	5.7^{b}	5.7^{b}	5.7^{b}	5.4^{c}	5.8^{a}
Pentanal	6.2^{e}	6.5^{d}	7.0^{b}	7.0^{b}	7.1^{a}	6.8^{c}	6.8^{c}	6.8^{c}	6.5^{d}	7.1^{a}
Hexanal	5.7^{d}	5.7^{d}	5.9^{c}	5.9^{c}	6.1^{a}	6.0^{b}	6.0^{b}	5.9^{c}	5.6^{e}	6.1^{a}
Heptanal	4.4^{e}	5.5^{c}	5.5^{c}	5.5^{c}	5.7^{a}	5.6^{b}	5.6^{b}	5.6^{b}	5.3^{d}	5.7^{a}
Octanal	5.4^{c}	5.4^{c}	5.3^{d}	5.3^{d}	5.5^{b}	5.6^{a}	5.6^{a}	5.4^{c}	5.2^{e}	5.5^{b}

Nonanal	5.9^{c}	5.8^{d}	5.8^{d}	5.8^{d}	5.9^{c}	6.2^{a}	6.2^{a}	6.0^{b}	5.9^{c}	5.9^{c}
Decanal	4.4^{c}	4.1^{e}	4.3^{d}	4.3^{d}	4.6^{b}	4.9^{a}	4.9^{a}	4.4^{c}	4.3^{d}	4.6^{b}
Propenal	5.2^{d}	5.1^{e}	5.3^{c}	5.3^{c}	5.4^{b}	5.5^{a}	5.5^{a}	5.1^{e}	4.9^{f}	5.4^{b}
2-Pentenal	4.7^{f}	4.8^{e}	5.1^{c}	5.1^{c}	5.3^{a}	5.2^{b}	5.2^{b}	5.0^{d}	4.6^{g}	5.3^{a}
2-Hexenal	4.7^{e}	4.6^{f}	5.0^{c}	5.0^{c}	5.2^{a}	5.1^{b}	5.1^{b}	4.8^{d}	4.6^{f}	5.2^{a}
2,4-Hexadienal	4.4^{c}	4.3^{d}	4.7^{a}	4.7^{a}	4.7^{a}	4.6^{b}	4.6^{b}	4.3^{d}	4.0^{e}	4.7^{a}
2-Heptenal	4.8^{e}	4.8^{e}	5.4^{b}	5.4^{b}	5.6^{a}	5.3^{c}	5.3^{c}	5.1^{d}	4.7^{f}	5.6^{a}
2-Octenal	4.7^{d}	4.4^{g}	4.8^{c}	4.8^{c}	4.9^{b}	5.1^{a}	5.1^{a}	4.6^{e}	4.5^{f}	4.9^{b}
2-Methyl-propanal	6.1^{c}	6.4^{a}	6.4^{a}	6.4^{a}	6.3^{b}	6.4^{a}	6.4^{a}	6.4^{a}	6.0^{d}	6.3^{ab}
3-Methyl-butanal	6.6^{e}	7.1^{b}	7.1^{b}	7.1^{b}	6.9^{c}	6.8^{d}	6.8^{d}	7.2^{a}	6.9^{c}	6.9^{c}
2-Methyl-butanal	6.1^{e}	6.6^{a}	6.5^{b}	6.5^{b}	6.4^{c}	6.3^{d}	6.3^{d}	6.6^{a}	6.3^{d}	6.4^{c}
Benzaldehyde	6.2^{e}	6.2^{e}	6.4^{d}	6.4^{d}	6.6^{b}	6.7^{a}	6.7^{a}	6.5^{c}	6.0^{f}	6.6^{b}
Benzeneacetaldehyde	5.6^{f}	5.9^{d}	6.2^{a}	6.2^{a}	6.2^{a}	6.1^{b}	6.1^{b}	6.0^{c}	5.8^{e}	6.2^{a}
Alcohols										
Methanol	5.0^{c}	5.1^{b}	5.2^{a}	5.2^{a}	5.1^{b}	5.2^{a}	5.2^{a}	5.1^{b}	5.1^{b}	5.1^{b}
Ethanol	6.6^{e}	6.9^{c}	7.0^{b}	7.0^{b}	7.3^{a}	6.9^{c}	6.9^{c}	6.7^{d}	6.6^{e}	7.3^{a}
1-Propanol	5.1^{d}	5.4^{a}	5.1^{d}	5.1^{d}	5.3^{b}	5.2^{c}	5.2^{c}	4.9^{e}	4.9^{e}	5.3^{b}
1-Butanol	5.9^{d}	6.1^{b}	6.2^{a}	6.2^{a}	6.0^{c}	5.9^{d}	5.9^{d}	6.0^{c}	5.8^{e}	6.0^{c}
1-Pentanol	6.3^{e}	6.5^{d}	7.0^{a}	7.0^{a}	7.0^{a}	6.8^{b}	6.8^{b}	6.8^{b}	6.6^{c}	7.0^{a}
Alcohols										
1-Hexanol	5.6^{d}	5.7^{c}	5.8^{b}	5.8^{b}	5.8^{b}	5.9^{a}	5.9^{a}	5.7^{c}	5.6^{d}	5.8^{b}
1-Heptanol	5.3^{c}	5.2^{d}	5.4^{b}	5.4^{b}	5.3^{c}	5.5^{a}	5.5^{a}	5.2^{d}	5.1^{e}	5.3^{c}
1-Octen-3-ol	5.0^{a}	5.0^{a}	5.0^{a}	5.0^{a}	4.9^{b}	4.4^{d}	4.4^{d}	5.0^{a}	4.7^{c}	4.9^{b}
2-Propanol	6.7^{d}	7.1^{a}	6.9^{c}	6.9^{c}	7.0^{b}	6.9^{c}	6.9^{c}	6.6^{e}	6.5^{f}	7.0^{b}
2-Butanol	5.3^{c}	5.6^{a}	5.4^{b}	5.4^{b}	5.4^{b}	5.6^{a}	5.6^{a}	5.3^{c}	5.3^{c}	5.4^{b}
2-Pentanol	6.4^{b}	6.8^{a}	6.4^{b}	6.4^{b}	6.4^{b}	6.1^{d}	6.1^{d}	6.3^{c}	5.9^{e}	6.4^{b}
2-Heptanol	7.3^{d}	7.8^{a}	7.7^{b}	7.7^{b}	7.2^{e}	6.6^{g}	6.6^{g}	7.4^{c}	7.1^{f}	7.2^{e}
2-Methyl-1-propanol	5.4^{b}	5.4^{b}	5.5^{a}	5.5^{a}	5.3^{c}	5.4^{b}	5.4^{b}	5.2^{d}	5.2^{d}	5.3^{c}
3-Methyl-3-buten-1-ol	5.0^{c}	5.1^{b}	5.1^{b}	5.1^{b}	5.0^{c}	5.2^{a}	5.2^{a}	4.8^{e}	4.9^{d}	5.0^{c}
3-Methyl-1-butanol	6.6^{c}	6.9^{a}	6.9^{a}	6.9^{a}	6.8^{b}	6.8^{b}	6.8^{b}	6.6^{c}	6.6^{c}	6.8^{b}
2-Methyl-1-butanol	5.8^{c}	6.0^{a}	5.9^{b}	5.9^{b}	5.8^{c}	5.8^{c}	5.8^{c}	5.7^{d}	5.6^{e}	5.8^{c}
2-Methyl-2-propanol	5.7^{a}	5.5^{c}	5.4^{d}	5.4^{d}	5.5^{c}	5.6^{b}	5.6^{b}	5.4^{d}	5.3^{e}	5.5^{c}
3-Methyl-2-butanol	5.3^{b}	5.5^{a}	5.3^{b}	5.3^{b}	5.3^{b}	5.1^{d}	5.1^{d}	5.2^{c}	5.1^{d}	5.3^{b}
3-Methyl-2-pentanol	4.9^{c}	5.1^{a}	5.0^{b}	5.0^{b}	4.7^{e}	4.7^{e}	4.7^{e}	4.8^{d}	4.7^{e}	4.7^{e}
4-Methyl-2-pentanol	5.0^{d}	5.3^{a}	5.2^{b}	5.2^{b}	5.0^{d}	5.2^{b}	5.2^{b}	5.1^{c}	5.0^{d}	5.0^{d}
Ketones										
2-Propanone	7.3^{a}	7.3^{a}	7.2^{b}	7.2^{b}	7.2^{b}	7.1^{c}	7.1^{c}	7.1^{c}	6.9^{d}	7.2^{b}
2-Butanone	7.0^{a}	7.0^{a}	6.9^{b}	6.9^{b}	6.9^{b}	6.9^{b}	6.9^{b}	7.0^{a}	6.8^{c}	6.9^{b}
2-Pentanone	8.6^{c}	8.8^{a}	8.7^{b}	8.7^{b}	8.5^{d}	8.3^{f}	8.3^{f}	8.4^{e}	8.2^{g}	8.5^{d}
3-Hexanone	5.8^{a}	5.8^{a}	5.6^{c}	5.6^{c}	5.6^{c}	5.4^{e}	5.4^{e}	5.7^{b}	5.5^{d}	5.6^{c}
2-Hexanone	6.8^{c}	7.2^{a}	7.0^{b}	7.0^{b}	6.7^{d}	6.5^{e}	6.5^{e}	6.7^{d}	6.4^{f}	6.7^{d}
4-Heptanone	5.9^{b}	6.0^{a}	5.8^{c}	5.8^{c}	5.7^{d}	5.3^{f}	5.3^{f}	6.0^{a}	5.6^{e}	5.7^{d}
3-Heptanone	4.4^{c}	4.5^{b}	4.6^{a}	4.6^{a}	4.5^{b}	4.4^{c}	4.4^{c}	4.5^{b}	4.4^{c}	4.5^{b}
2-Heptanone	8.2^{c}	8.5^{a}	8.5^{a}	8.5^{a}	8.1^{d}	7.7^{f}	7.7^{f}	8.3^{b}	8.0^{e}	8.1^{d}
3-Octanone	5.3^{b}	5.3^{b}	5.3^{b}	5.3^{b}	5.1^{c}	5.1^{c}	5.1^{c}	5.4^{a}	5.1^{c}	5.1^{c}
2-Octanone	6.3^{d}	6.7^{b}	6.8^{a}	6.8^{a}	6.2^{e}	6.0^{f}	6.0^{f}	6.5^{c}	6.2^{e}	6.2^{e}
4-Nonanone	4.9^{a}	4.6^{c}	4.6^{c}	4.6^{c}	4.5^{d}	4.6^{c}	4.6^{c}	4.8^{b}	4.4^{e}	4.5^{d}
2-Nonanone	7.1^{d}	7.5^{b}	7.6^{a}	7.6^{a}	7.3^{c}	7.0^{e}	7.0^{e}	7.5^{b}	7.1^{d}	7.3^{c}

3-Buten-2-one	5.7^{a}	5.6^{b}	5.6^{b}	5.6^{b}	5.7^{a}	5.7^{a}	5.7^{a}	5.6^{b}	5.5^{c}	5.7^{a}
3-Penten-2-one	5.3^{c}	5.5^{a}	5.4^{b}	5.4^{b}	5.3^{c}	5.0^{f}	5.0^{f}	5.2^{d}	5.1^{e}	5.3^{c}
5-Hexen-2-one	5.6^{b}	5.9^{a}	5.5^{c}	5.5^{c}	5.5^{c}	5.5^{c}	5.5^{c}	5.4^{d}	5.3^{e}	5.5^{c}
6-Hepten-2-one	5.3^{d}	5.7^{a}	5.6^{b}	5.6^{b}	5.3^{d}	4.9^{f}	4.9^{f}	5.4^{c}	5.1^{e}	5.3^{d}
5-Hepten-2-one	5.4^{d}	6.0^{a}	5.2^{e}	5.2^{e}	5.4^{d}	4.5^{f}	4.5^{f}	5.6^{c}	5.7^{b}	5.4^{d}
1-Octen-3-one	5.0^{e}	5.0^{e}	5.3^{b}	5.3^{b}	5.5^{a}	5.2^{c}	5.2^{c}	5.1^{d}	4.9^{f}	5.5^{a}
8-Nonen-2-one	5.6^{e}	6.2^{b}	6.3^{a}	6.3^{a}	5.7^{d}	5.4^{f}	5.4^{f}	6.1^{c}	5.7^{d}	5.7^{d}
3-Hydroxy-2-butanone	5.0^{b}	4.8^{d}	4.9^{c}	4.9^{c}	4.5^{e}	5.1^{a}	5.1^{a}	4.8^{d}	4.4^{f}	4.5^{e}
2,3-Butanedione	6.5^{d}	6.9^{a}	6.8^{b}	6.8^{b}	6.6^{c}	6.4^{e}	6.4^{e}	6.8^{b}	6.5^{d}	6.6^{c}
2,3-Pentanedione	5.2^{d}	5.4^{c}	5.6^{a}	5.6^{a}	5.6^{a}	5.5^{b}	5.5^{b}	5.4^{c}	5.2^{d}	5.6^{a}
2,3-Hexanedione	4.3^{c}	4.0^{e}	4.6^{a}	4.6^{a}	4.2^{d}	4.4^{b}	4.4^{b}	4.4^{b}	4.3^{c}	4.2^{d}
2,3-Octanedione	5.0^{d}	5.0^{d}	5.4^{c}	5.4^{c}	5.6^{a}	5.5^{b}	5.5^{b}	5.4^{c}	5.0^{d}	5.6^{a}
3-Methyl-2-butanone	5.9^{a}	5.8^{b}	5.7^{c}	5.7^{c}	5.6^{d}	5.6^{d}	5.6^{d}	5.7^{c}	5.7^{c}	5.6^{d}
4-Methyl-2-pentanone	5.6^{b}	5.2^{e}	5.7^{a}	5.7^{a}	5.5^{c}	5.3^{d}	5.3^{d}	5.6^{b}	5.5^{c}	5.5^{c}
2-Methyl-3-pentanone	4.8^{a}	4.7^{b}	4.7^{b}	4.7^{b}	4.7^{b}	4.7^{b}	4.7^{b}	4.8^{a}	4.7^{b}	4.7^{b}
Ketones										
3-Methyl-2-pentanone	6.5^{a}	6.4^{b}	6.4^{b}	6.4^{b}	6.3^{c}	6.2^{d}	6.2^{d}	6.4^{b}	6.4^{b}	6.3^{c}
5-Methyl-2-hexanone	5.2^{b}	5.5^{a}	5.5^{a}	5.5^{a}	5.0^{c}	4.8^{e}	4.8^{e}	5.2^{b}	4.9^{d}	5.0^{c}
3-Methyl-2-heptanone	5.5^{c}	5.8^{a}	5.7^{b}	5.7^{b}	5.4^{d}	5.0^{f}	5.0^{f}	5.7^{b}	5.3^{e}	5.4^{d}
6-Methyl-3-heptanone	4.5^{b}	4.4^{c}	4.4^{c}	4.4^{c}	4.1^{e}	3.4^{f}	3.4^{f}	4.6^{a}	4.3^{d}	4.1^{e}
6-Methyl-2-heptanone	4.7^{d}	4.9^{b}	5.0^{a}	5.0^{a}	4.8^{c}	4.9^{b}	4.9^{b}	4.8^{c}	4.6^{e}	4.8^{c}
5-Methyl-2-heptanone	4.6^{d}	5.1^{a}	5.1^{a}	5.1^{a}	4.7^{c}	4.5^{e}	4.5^{e}	4.9^{b}	4.4	4.7^{c}
Cyclopentanone	4.7^{c}	4.8^{b}	4.6^{d}	4.6^{d}	4.5^{e}	5.0^{a}	5.0^{a}	4.8^{b}	4.5^{e}	4.5^{e}
1-Phenyl-ethanone	5.4^{c}	5.5^{b}	5.5^{b}	5.5^{b}	5.5^{b}	5.7^{a}	5.7^{a}	5.5^{b}	5.4^{c}	5.5^{b}
Esters										
Methyl acetate	4.6^{e}	5.0^{a}	4.9^{b}	4.9^{b}	4.8	4.9^{b}	4.9^{b}	4.7^{d}	4.4^{f}	4.8^{c}
Methyl butanoate	5.5^{d}	5.9^{a}	5.8^{b}	5.8^{b}	5.6^{c}	5.5^{d}	5.5^{d}	5.4^{e}	5.2^{f}	5.6^{c}
Methyl 3-methyl-butanoate	4.2^{b}	4.3^{a}	4.3^{a}	4.3^{a}	4.1^{c}	4.3^{a}	4.3^{a}	3.9^{d}	3.3^{e}	4.1^{c}
Methyl 2-methyl-butanoate	4.2^{b}	4.2^{b}	4.3^{a}	4.3^{a}	4.2^{b}	4.3^{a}	4.3^{a}	4.0^{c}	3.8^{d}	4.2^{b}
Methyl pentanoate	4.4^{e}	4.8^{b}	4.9^{a}	4.9^{a}	4.9^{a}	4.5^{d}	4.5^{d}	4.8^{b}	4.6^{c}	4.9^{a}
Methyl hexanoate	5.8^{d}	6.1^{a}	6.1^{a}	6.1^{a}	6.0^{b}	5.9^{c}	5.9^{c}	5.9^{c}	5.8^{d}	6.0^{b}
Methyl octanoate	5.2^{c}	5.4^{a}	5.4^{a}	5.4^{a}	5.4^{a}	5.4^{a}	5.4^{a}	5.3^{b}	5.2^{c}	5.4^{a}
Methyl benzoate	5.4^{c}	5.4^{c}	5.5^{b}	5.5^{b}	5.6^{a}	5.6^{a}	5.6^{a}	5.4^{c}	5.4^{c}	5.6^{a}
Ethyl acetate	5.2^{d}	5.8^{a}	5.8^{a}	5.8^{a}	5.8^{a}	5.8^{a}	5.8^{a}	5.6^{b}	5.5^{c}	5.8^{a}
Ethyl propanoate	3.6^{d}	3.9^{a}	3.7^{c}	3.7^{c}	3.7^{c}	3.8^{b}	3.8^{b}	2.9^{f}	3.0^{e}	3.7^{c}
Ethyl 2-methyl-propionate	4.0^{c}	4.2^{a}	4.2^{a}	4.2^{a}	4.1^{b}	4.1^{b}	4.1^{b}	3.9^{d}	3.8^{e}	4.1^{b}
Ethyl butanoate	6.0^{c}	6.2^{a}	6.2^{a}	6.2^{a}	6.0^{c}	6.1^{b}	6.1^{b}	5.9^{d}	5.9^{d}	6.0^{c}
Ethyl 2-butenoate	4.4^{a}	4.3^{b}	4.1^{d}	4.1^{d}	4.2^{c}	4.3^{b}	4.3^{b}	4.3^{b}	4.3^{b}	4.2^{c}
Ethyl 2-methyl-butanoate	3.2^{f}	4.0^{b}	4.1^{a}	4.1^{a}	3.8^{c}	3.5^{d}	3.5^{d}	3.4^{e}	0.0^{g}	3.8^{c}
Ethyl 3-methyl-butanoate	3.6^{d}	4.3^{a}	4.3^{a}	4.3^{a}	4.3^{a}	4.2^{b}	4.2^{b}	4.1^{c}	3.6^{d}	4.3^{a}
Ethyl pentanoate	3.9^{d}	4.3^{b}	4.4^{a}	4.4^{a}	4.3^{b}	4.3^{b}	4.3^{b}	4.2^{c}	3.4^{e}	4.3^{b}
Ethyl hexanoate	5.9^{e}	6.3^{a}	6.3^{a}	6.3^{a}	6.2^{b}	6.2^{b}	6.2^{b}	6.1^{c}	6.0^{d}	6.2^{b}
Ethyl heptanoate	3.6^{e}	4.2^{a}	4.2^{a}	4.2^{a}	4.1^{b}	4.2^{a}	4.2^{a}	3.9^{c}	3.8^{d}	4.1^{b}
Ethyl octanoate	4.4^{f}	4.9^{c}	4.9^{c}	4.9^{c}	5.0^{b}	5.1^{a}	5.1^{a}	4.8^{d}	4.6^{e}	5.0^{g}
Esters										
Propyl acetate	4.3^{b}	4.3^{b}	4.4^{a}	4.4^{a}	4.4^{a}	4.4^{a}	4.4^{a}	4.4^{a}	4.3^{b}	4.4^{a}
Propyl butanoate	4.3^{c}	4.6^{a}	4.4^{b}	4.4^{b}	4.3^{c}	4.2^{d}	4.2^{d}	4.2^{d}	3.9^{e}	4.3^{c}
Propyl hexanoate	4.1^{c}	3.9^{d}	4.2^{b}	4.2^{b}	4.2^{b}	4.3^{a}	4.3^{a}	3.9^{d}	3.8^{e}	4.2^{b}

Butyl acetate	5.0^{d}	5.0^{d}	5.3^{b}	5.3^{b}	5.1^{c}	5.8^{a}	5.8^{a}	5.0^{d}	5.1^{c}	5.1^{c}
Butyl butanoate	3.7^{c}	4.1^{a}	4.1^{a}	4.1^{a}	3.6^{d}	2.9^{e}	2.9^{e}	3.9^{b}	0.0^{f}	3.6^{d}
Pentyl acetate	3.5^{d}	3.7^{c}	4.0^{b}	4.0^{b}	4.2^{a}	3.7^{c}	3.7^{c}	3.7^{c}	3.1^{e}	4.2^{a}
2-Pentyl butanoate	4.4^{b}	4.6^{a}	3.8^{e}	3.8^{e}	4.1^{d}	4.3^{c}	4.3^{c}	3.7^{f}	3.8^{e}	4.1^{d}
Hexyl acetate	4.0^{c}	3.7^{e}	3.9^{d}	3.9^{d}	3.9^{d}	4.2^{b}	4.2^{b}	4.3^{a}	3.7^{e}	3.9^{d}
3-Methyl-butyl acetate	4.8^{f}	5.4^{a}	5.2^{c}	4.5^{g}	5.3^{b}	5.1^{d}	4.2^{i}	5.3^{b}	4.9^{e}	4.3^{h}
2-Methyl-butyl acetate	4.4^{d}	4.6^{c}	4.6^{c}	4.0^{g}	4.7^{b}	4.8^{a}	4.1^{f}	4.6^{c}	4.3^{e}	4.1^{f}
1-Methyl-propyl butanoate	3.9^{e}	4.2^{c}	4.2^{c}	5.2^{a}	3.8^{f}	4.0^{d}	5.1^{b}	3.6^{g}	3.5^{h}	5.3^{i}
2-Methyl-propyl butanoate	5.0^{c}	5.1^{b}	5.2^{a}	4.6^{g}	4.9^{d}	5.0^{c}	4.8^{e}	4.9^{d}	4.7^{f}	4.7^{f}
Isopropyl butanoate	4.4^{c}	4.6^{a}	4.5^{b}	4.2^{e}	4.3^{d}	4.2^{e}	4.0^{f}	4.2^{e}	3.8^{g}	3.8^{g}
2-Methyl-propyl acetate	3.8^{f}	4.1^{d}	4.0^{e}	5.2^{a}	4.1^{d}	4.1^{d}	5.0^{b}	4.1^{d}	3.8^{f}	4.9^{c}
Esters										
3-Methyl-butyl butanoate	5.2^{c}	5.3^{b}	5.4^{a}	5.4^{a}	5.2^{c}	5.3^{b}	5.3^{b}	5.1^{d}	5.0^{e}	5.2^{c}
2-Methyl-butyl butanoate	4.8^{c}	4.9^{b}	4.9^{b}	4.9^{b}	4.8^{c}	5.1^{a}	5.1^{a}	4.7^{d}	4.4^{e}	4.8^{c}
Sulfur compounds										
Methanethiol	4.7^{d}	4.7^{d}	5.0^{c}	5.0^{c}	5.4^{a}	5.1^{b}	5.1^{b}	4.7^{d}	4.4^{e}	5.4^{a}
Dimethyl-sulfide	4.2^{a}	4.0^{c}	4.1^{b}	4.1^{b}	3.8^{e}	4.2^{a}	4.2^{a}	4.0^{c}	3.9^{d}	3.8^{e}
Dimethyl-disulfide	6.6^{c}	6.5^{d}	6.7^{b}	6.7^{b}	6.9^{a}	6.9^{a}	6.9^{a}	6.7^{b}	6.6^{c}	6.9^{a}
Dimethyl-trisulfide	4.8^{c}	4.6^{e}	4.8^{c}	4.8^{c}	5.1^{b}	5.5^{a}	5.5^{a}	4.7^{d}	4.5^{f}	5.1^{b}
2,4-Dithiapentane	5.6^{b}	5.5^{c}	5.5^{c}	5.5^{c}	5.4^{d}	5.2^{f}	5.2^{f}	5.9^{a}	4.8^{g}	5.4^{e}
Methional	4.1^{f}	4.3^{e}	4.6^{c}	4.0^{g}	4.8^{b}	5.0^{a}	4.3^{e}	4.5^{d}	3.6^{h}	4.0^{g}
S-methyl thioacetate	5.1^{d}	5.3^{c}	5.6^{a}	4.6^{g}	5.4^{b}	5.0^{e}	5.0^{e}	5.4^{b}	5.3^{c}	4.8^{f}
Thiophene	5.1^{d}	5.3^{c}	5.3^{c}	5.6^{a}	5.3^{c}	5.6^{a}	5.0^{e}	5.3^{c}	5.1^{d}	5.4^{b}
2-Methyl-tetrahydro-thiophen-3-one	4.9^{c}	4.8^{d}	4.7^{e}	5.3^{b}	4.9^{c}	4.9^{c}	5.6^{a}	4.8^{d}	4.8^{d}	5.3^{b}
Methyl isothiocyanate	5.1^{e}	4.9^{f}	5.9^{b}	4.7^{h}	6.4^{a}	5.8^{c}	4.9^{f}	5.2^{d}	4.8^{g}	4.9^{f}
Furans										
2-Methyl-furan	4.7^{f}	4.6^{g}	4.9^{d}	5.9^{b}	4.9^{d}	4.8^{e}	5.8^{c}	4.8^{e}	4.6^{g}	6.4^{a}
2-Ethyl-furan	5.3^{c}	5.2^{d}	5.5^{b}	4.9^{f}	5.6^{a}	5.6^{a}	4.8^{g}	5.3^{c}	5.1^{e}	4.9^{f}
2-Propyl-furan	4.6^{e}	4.5^{f}	4.6^{e}	5.5^{b}	4.7^{d}	4.8^{c}	5.6^{a}	4.5^{f}	4.5^{f}	5.6^{a}
3-Methyl-furan	4.8^{b}	4.7^{c}	4.8^{b}	4.6^{d}	4.7^{c}	4.6^{d}	4.8^{b}	4.9^{a}	4.6^{d}	4.7^{c}
2,4-Dimethyl-furan	4.6^{f}	4.8^{d}	4.9^{c}	4.3^{i}	4.5^{g}	4.7^{e}	4.4^{h}	5.2^{a}	5.0^{b}	3.9^{j}
2,5-Dimethyl-furan	4.9^{a}	4.4^{f}	4.3^{g}	4.8^{b}	3.9^{h}	4.4^{f}	4.6^{d}	4.5^{e}	4.4^{f}	4.7^{c}
Furfural	4.8^{d}	4.5^{g}	5.1^{a}	4.9^{c}	4.7^{e}	5.0^{b}	4.7^{e}	4.6^{f}	4.5^{g}	4.5^{g}

[*]Only VOC that showed variation ($P \leq 0.05$) between samples. The data are the means of three independent experiments, and values in the same row followed by different lowercase letters (a to k) differs significantly values ± standard deviations for three batches of each type of cheese, analysed in duplicate. Row with different superscript letters are significantly different ($P \leq 0.05$).

[§]Slice of each cheese was cut into nine sub-blocks identified by the letters A - I. Sub-blocks A, D, and G, and sub-blocks C, F and I were collected from top and bottom surface region, respectively, whereas sub-blocks B and H from inner side region, and sub-block E from the core. The whole slice was the control. Further details were reported in the Material and Methods and in Fig. 1.
