# Supplementary material for: Spatial Distribution of the Metabolically Active Microbiota within Italian PDO Ewes' Milk Cheeses
Source: PLoS One. 2016 Apr 13;11(4):e0153213. doi: 10.1371/journal.pone.0153213 (PMC4830609; doi:10.1371/journal.pone.0153213)
Supplement: S4 Table — (PDF) [file pone.0153213.s007.pdf]

S4 Table. Observed diversity and estimated sample coverage of 16S rRNA sequencing analysis of Fiore Sardo, Pecorino Siciliano and Pecorino Toscano cheeses.

Cheese	Sample	No. of reads	No. of OTUs	Chao1 richness	Shannon diversity index
Fiore Sardo	Control*	8379	17	20	1.07
	A	5112	19	24	1.02
	D	4169	18	39	1.03
	G	4009	15	15.5	1.16
	B	4626	15	15.2	0.99
	E	6488	19	19.6	0.93
	H	3706	16	17	0.98
	C	4065	17	20	1.01
	F	5540	19	25	1.07
	I	4196	13	14.5	1.03
Pecorino Siciliano	Control*	3110	31	31.2	1.72
	A	2763	18	24	1.24
	D	3070	24	26	1.43
	G	4487	23	26.3	1.32
	B	2357	21	21.6	1.20
	E	4264	23	24	1.05
	H	3338	24	24	1.19
	C	4124	23	24.4	1.24
	F	3430	27	28.2	1.56
	I	3989	21	24	1.42
Pecorino Toscano	Control*	4923	15	15.2	0.49
	A	4184	16	16.3	0.61
	D	6220	20	20.3	0.74
	G	6342	18	18.3	0.62
	B	4652	13	16	0.27
	E	9402	9	9	0.17
	H	6630	14	15.5	0.28
	C	5749	14	17	0.34
	F	5062	10	14	0.30
	I	4476	17	24	0.34

Abbreviations: OTU, operational taxonomic unit; Chao1 richness and Shannon diversity were calculated with USEARCH based classifier.

*Slice of each cheese was cut into nine sub-blocks identified by the letters A - I. Sub-blocks A, D, and G, and sub-blocks C, F and I were collected from top and bottom surface region, respectively, whereas sub-blocks B and H from inner side region, and sub-block E from the core. The whole slice was the control. Further details were reported in the Material and Methods and in Fig. 1.
